# Performance and Stability Analysis of Selected Durum Wheat Genotypes Differing in Their Kernel Characteristics

**DOI:** 10.3390/plants12142664

**Published:** 2023-07-16

**Authors:** R. Al-Sayaydeh, M. J. Shtaya, T. Qubbaj, M. K. Al-Rifaee, M. A. Alabdallah, O. Migdadi, I. A. Gammoh, A. M. Al-Abdallat

**Affiliations:** 1 Department of Agriculture Sciences, Faculty of Shoubak College, Al-Balqa Applied University, Al-Salt 19117, Jordan; 2 Department of Plant Production and Protection, Faculty of Agriculture and Veterinary Medicine, An-Najah National University, Nablus P.O. Box 707, Palestine; mshtaya@najah.edu (M.J.S.); tqubbaj@najah.edu (T.Q.); 3 National Agricultural Research Center (NARC), Amman 19381, Jordan; m.rifaee@narc.gov.jo (M.K.A.-R.); mamk_aaa@yahoo.com (M.A.A.); himigdadi@yahoo.com (O.M.); 4 Department of Horticulture and Crop Science, School of Agriculture, The University of Jordan, Amman 11942, Jordan; i.gammoh@ju.edu.jo

**Keywords:** AMMI analysis, drought, GGE biplot, grain yield, kernel length, salinity, thousand kernel weight, *Triticum* *turgidum* L. subsp. *durum*

## Abstract

Breeding of high-yielding and stable durum wheat varieties with improved kernel characteristics is needed for dry regions around the globe. The aim of this study was to investigate the performance and stability of eight durum wheat genotypes varying in their kernel characteristics across 15 contrasting environments. The tested material included three recombinant inbred lines (NUR-072, NUR-106 and NUR-238) derived from a cross between Norsi, a Jordanian landrace with special kernel characteristics and UC1113 *Yr36+Gpc-B1*, an elite line from USA. Field trials were carried out for three constitutive growing seasons under rainfed conditions, except for three environments where supplementary irrigation was provided. After the harvest, grain yield (GY), total yield (TW), and harvest index (HI) were recorded. Additionally, several kernel-related traits, including thousand kernel weight (TKW), kernel area (KA), kernel width (KW), kernel length (KL), kernel circularity (KC), and kernel length–width ratio (KL:KW) were evaluated. Analysis of variance for all tested traits revealed high significant variations (*p ≤ 0.01*) between the genotype (except for TW) and the genotype × environment (G × E) interaction. Genotype effect contributed to substantial percentage of variation (>75%) for KA, KL, KC and KL:KW, whereas KW showed a lower percentage similar to GY. Regarding the G × E effect, explained variation was highest for the TW (67.79%), and lowest for KL (6.47%). For GY, Norsi produced significantly the lowest mean value (249.99 g.m^−2^) while, Bolenga produced the highest mean value (377.85 g.m^−2^) although no significant differences were observed with the remaining genotypes. On the other hand, Norsi, NUR-072 and NUR-106 showed best performance for TKW and kernel-related traits with NUR-106 producing the highest mean value for KL (9.07 mm). The GGE biplot and AMMI analysis of GY identified Bolenga, Um Qais and NUR-106 as good performers across several environments, while Norsi exhibited the poorest performance. For TKW, Norsi was the best performer across different environments followed by NUR-106, which showed excellent performance under irrigated and saline conditions. For stability analysis, NUR-106 emerged as the most stable genotype in this study for GY and several kernel-related traits, particularly for KL and KC. In conclusion, the results of this study offer valuable insights for durum wheat breeders seeking to develop high-yielding and stable varieties with special kernel characteristics suitable for cultivation in dry areas.

## 1. Introduction

Durum wheat (*Triticum turgidum* L. subsp. *durum* (Desf.) van Slageren) is an important cereal crop grown on ~17.7 million hectares worldwide with an estimated global production of 39.5 million tons [1]. Originally domesticated in dry areas of the Levant, durum wheat is well adapted to harsh environments and exhibits significant tolerance to drought and heat conditions [2]. It is the crop of choice in many countries along the Mediterranean Rim and is a major constituent of the Mediterranean diet. Durum wheat is typically used in many products including pasta, bourghul and couscous, as well as in several dishes in Mediterranean cuisine such as Gofio and Freekeh, and different types of flatbread [3]. Nowadays, there is a global trend pointing to increasing demand for durum wheat products as they are a major contributor to healthy, balanced, and nutritious diets [4,5].

Durum wheat is facing major challenges in dry areas that will affect its productivity, with expected negative impacts from climate change and associated conditions [6]. Future climate change models predict that durum wheat production areas will be affected drastically, especially in the Mediterranean region. Reduction in durum wheat yield is expected due to drought, heat, and saline conditions as well as shorter growing seasons [7]. Furthermore, durum wheat is considered a sensitive plant to saline conditions with a clear reduction in grain yield (GY) under such conditions [8]. Therefore, there is an urgent need to produce new durum wheat varieties that have enhanced tolerance to dry and saline conditions.

Over the last 50 years, most of the progress in releasing stress-tolerant durum wheat varieties has been achieved primarily through conventional or traditional breeding methods [9]. The improvement of GY has been primarily attributed to manipulating selected yield-component traits with a focus on plant height, flowering time, and harvest index [10,11]. However, genetic gains in wheat yield have substantially slowed down in recent years due to the lack of ‘breakthrough’ germplasm and the use of conventional breeding approaches [6]. Additionally, genetic gain associated with GY was further reduced due to climate change and extreme weather conditions such drought and heat [12]. To meet the increasing demand and to cope with climate change, the present annual yield increase must be raised from the current level of less than 1% to at least 1.7% [13]. In addition, modern breeding approaches have led to reduce genetic diversity in major durum wheat cultivated areas [14]. Recently, resilient durum wheat production systems that are based on modern breeding approaches and the adoption of new management technologies was proposed [13].

Kernel-related traits were extensively and intensively explored for wheat improvement [15,16]. Several studies on wheat kernel weight (often expressed as thousand kernel weight (TKW)) and other kernel-associated traits have emphasized their importance in yield improvement [17,18]. Genetic improvement of kernel size and shape is considered central to developing high-yielding wheat cultivars with improved commercial potential and adaption to diverse environments [19,20,21]. On the other hand, kernel-related traits are considered unstable and are severely influenced by environmental stresses such as heat, drought, and salinity [22,23]. Furthermore, numerous genetic studies have shown that variation in kernel dimensions is mainly controlled by different mechanisms and exhibits a strong genotype by environment (G × E) effect [16,24]. Therefore, it is important to understand the role of kernel-related traits in wheat plant under a range of different environments and in particular under dry areas.

The main objective of this study was to evaluate the performance and yield stability of eight selected durum wheat genotypes differing in their kernel characteristics. The durum wheat material was tested in 15 contrasting environments across Jordan, a country in the eastern Mediterranean basin characterized by its dry conditions. The relationship between kernel-related traits, in particular TKW and GY, was assessed and genotypes demonstrating high stability for the targeted traits were identified. The main hypothesis question of this study is whether kernel-related traits can contribute to the breeding of high-yielding and stable durum wheat varieties suitable for dry regions. The identification of stable and high-yielding durum wheat genotypes with improved kernel characteristics will facilitate their adoption in dry areas.

## 2. Results

### 2.1. Field Data

Weather data recorded for each environment, including rainfall and average monthly maximum and minimum temperatures, are shown in Table S1. The 15 environments received varying amounts of rainfall ranging from 102.0 mm in KD during the 2021 season to 742 mm in JU during the 2020 growing season. Five of the testing environments received a total rainfall lower than the long-term average, while 10 environments received a total rainfall higher than the long-term average. During 2020 season, all tested locations received a total precipitation higher than the long-term average, while in 2021, all tested locations received a total rainfall lower than the long-term average except for JU (Table S1). Additionally, rain ceased in April 2021 across all locations (except JU), imposing terminal drought conditions in rainfed environments where supplementary irrigation was not provided. These results indicate that the 2020 season was considered a wet season, while 2021 season was considered a dry season. Regarding the average monthly maximum and minimum temperatures, January was considered the coldest month across all tested environments, while the highest maximum and minimum temperature averages were recorded in June (Table S1). The lowest average minimum and maximum temperatures were recorded in January 2022 in the SK station, whereas the highest average maximum temperature was recorded in KD in May 2021, and the highest average minimum temperature was recorded in May 2021 in the KD station (Table S1). During the 2021 season, January was considerably warmer across locations when compared to its data in other growing seasons (Table S1). The overall conclusion indicates that the 2021 season was drier and warmer than usual, while 2020 season was considered a wet season and the 2022 season showed lower average temperatures in March and rainfall amounts near the overall averages.

The combined ANOVA revealed highly significant differences (*p* ≤ 0.01) for all tested traits among environment, genotype (except for TW) and G × E interaction (Table S2). Grand mean values, broad-sense heritability (*H*^2^) and the coefficient of variation (CV) for the studied traits across the tested environments are given in Table S2. The CV values ranged from 2.31% for KW to 11.37% for GY. For the *H*^2^ values, TW produced the lowest estimates (0.36), while TKW, KA, KL, KC and KL:KW produced *H*^2^ values > 0.95. For GY and HI, the *H*^2^ values were 0.85 and 0.90, respectively. The grand mean for GY across all tested environments was 336.27 g.m^−2^, whereas, for TKW, the grand mean was 38.32 g (Table S2). The variation explained by the genotype effect ranged from 2.85% for TW to 85.92% for KL (Table S2). The percentage of variation attributed to the genotype effect was 23.46% for GY and 58.01% for TKW (Table S2). On the other hand, kernel-related traits, including KA, KL, KC and KL:KW, showed high percentages of variation explained by the genotype effect (exceeding 75%), while KW (23.59%) showed a similar variation percentage to the GY. The variation explained by the G × E effect was highest for the TW (67.79%), followed by GY (57.74%) and KW (51.16%) (Table S2). For KL, the G × E effect accounted for 6.47% only from the total variance, while for TKW, it accounted for 33.60%.

**Figure 1:**
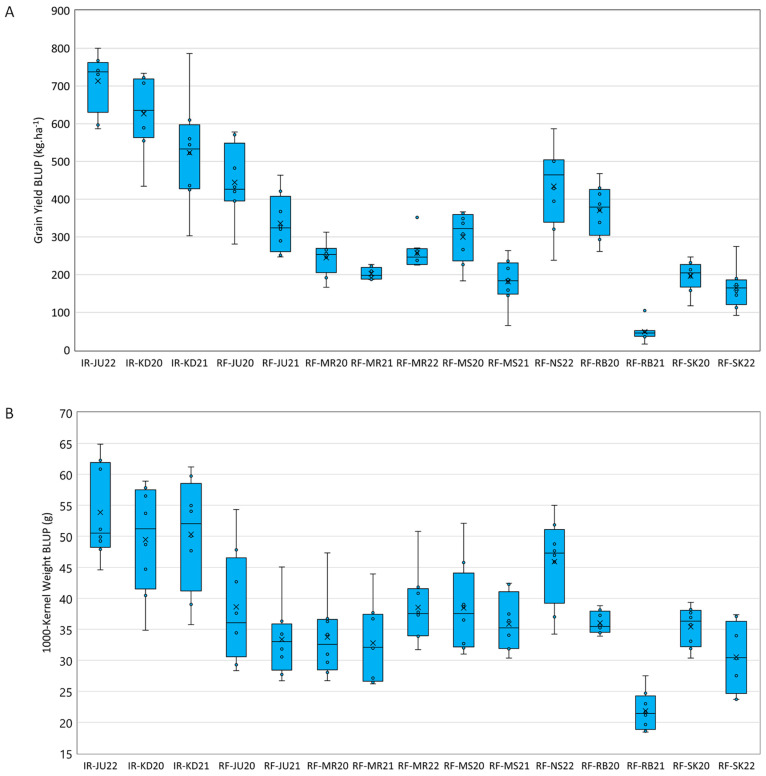
Boxplots of BLUPs of eight durum wheat genotype for grain yield (**A**) and thousand kernel weight (**B**) tested across 15 tested environments.

Significant differences (*p*  ≤  0.001) for all tested traits were observed due to the G × E effect (Table S2). The values of BLUPs of the durum wheat genotypes for all recorded traits in each environment and overall combined analysis are given in Table S3 and their distributions for GY and TKW across tested environments are shown in Figure 1. As expected, high means for GY BLUPs were observed in rainfed environments with continuous supplementary irrigation (IR-JU22, IR-KD20, and IR-KD21) with IR-JU22 environment producing the highest mean value, while RF-RB21 exhibited the lowest mean values (Figure 1A and Table S3). UC1113 *Yr36+Gpc-B1* recorded the lowest mean value (16.76 g.m^−2^) in RF-RB21, while NUR-072 produced the highest mean value (800.92 g.m^−2^) in IR-JU22 (Table S3). For TKW, the IR-JU22 environment produced the highest mean value, while the lowest mean values were observed for RF-RB21 (Figure 1B). Norsi recorded the highest mean value (64.66 g) in IR-JU22, while UC1113 *Yr36+Gpc-B1* produced the lowest mean value (18.10 g) in RF-RB21 (Table S3). For KL, NUR-106 produced the highest BLUPs values in all tested environments except in RF-JU21 and RF-SK22, while Um Qais produced the lowest BLUPs values except in RF-RB21 and RF-SK22 (Table S3).

**Figure 2:**
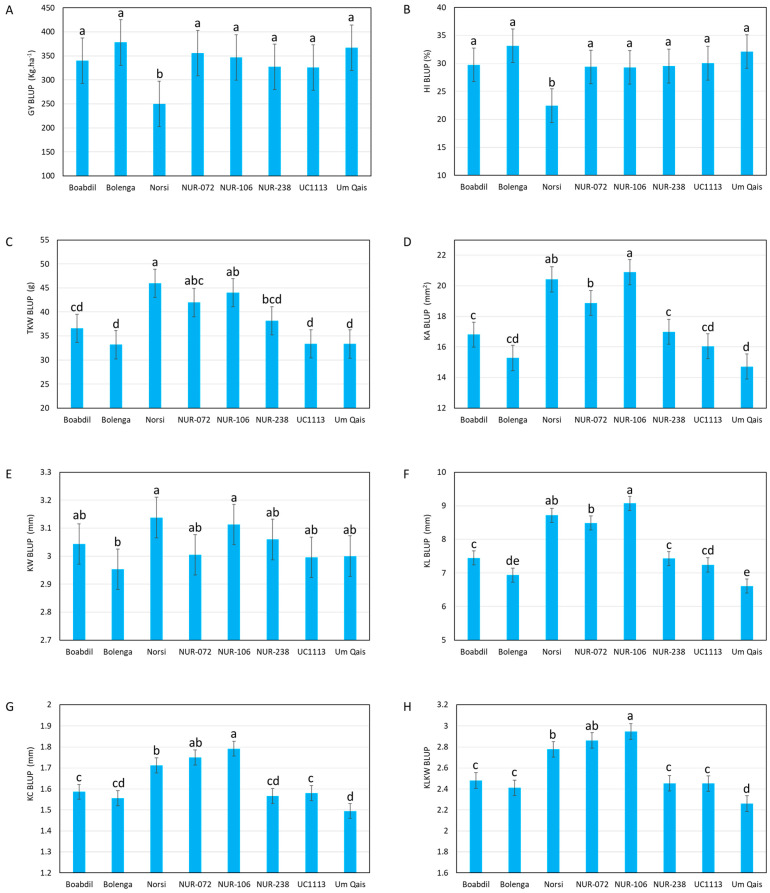
Means of the BLUPs of eight durum wheat genotype combined over 15 tested environments for grain yield (GY) (**A**), harvest index (HI) (**B**), thousand kernel weight (TKW) (**C**), kernel area (KA) (**D**), kernel width (KW) (**E**), kernel length (KL) (**F**), kernel circularity (KC) (**G**) and kernel length to kernel width ration (KL:KW) (**H**). Means followed by different letters are significantly different according to the least significant difference (LSD) test at *p* ≤ 0.05.

For the genotypic effect, the mean values of the GY BLUPs for the tested genotypes ranged from 249.99 g.m^−2^ for Norsi landrace to 377.85 g.m^−2^ recorded in Bolenga (Figure 2A). Significant differences were observed in the mean values of GY between Norsi, which had the lowest mean value, and the other tested genotypes, where no significant differences were observed among them (Figure 2A). Similar to GY, Norsi significantly exhibited the lowest mean value for HI among the tested genotypes (Figure 2B). For kernel-related traits, Norsi, NUR-072, NUR-106 genotypes showed significant differences compared to the other tested genotypes for TKW (except for NUR-238 compared to NUR-072 and NUR-106), KA, KL, KC and KL:KW, while for KW, significant differences were found between Bolenga compared to Norsi as well as NUR-106 (Figure 2C–H).

**Figure 3:**
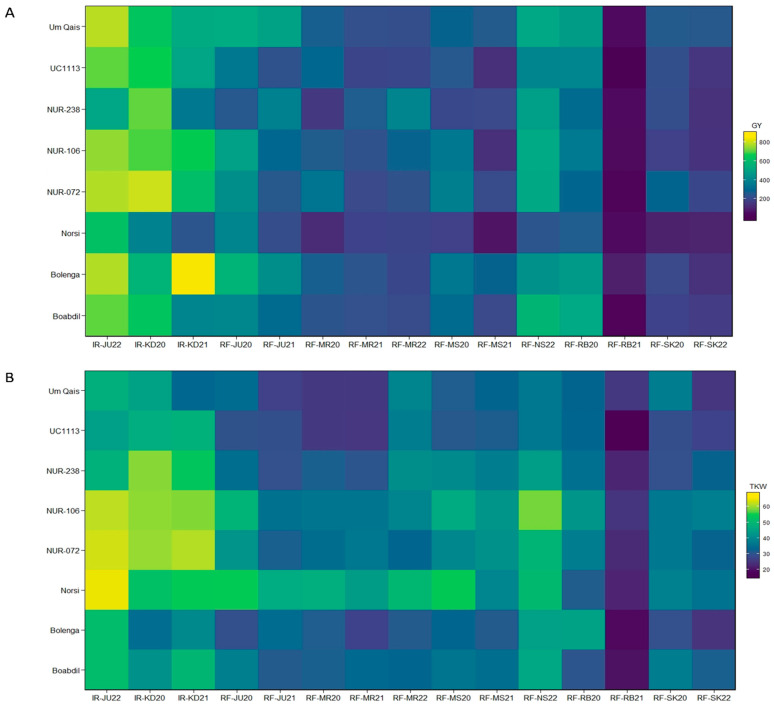
Heatmaps for eight durum wheat genotype performance across 15 tested environments for grain yield (GY) (**A**), and thousand kernel weight (TKW) (**B**).

Heatmaps were constructed to analyze the performance of genotypes across the tested environments for the GY and TKW traits (Figure 3). All tested genotypes were severely affected by the drought conditions in the 2021 season with the greatest effect observed in RF-RB21. However, rainfed environments with supplementary irrigation (IR-JU22, IR-KD21, and IR-KD22) exhibited high GY and TKW (Figure 3). Norsi performed poorly in terms of GY performance across all tested environments compared to other tested genotypes. However, Norsi was among the best genotypes for TKW performance (Figure 3). Regarding kernel-related traits, NUR-106 showed excellent performance across several environments for KA, KL, KC, and KL:KW (Figure S1). The kernels of selected genotypes grown under supplementary irrigation (IR-JU22) and rainfed conditions (RF-NS22) are shown in Figure S2.

### 2.2. Correlation and Cluster Analysis

**Figure 4:**
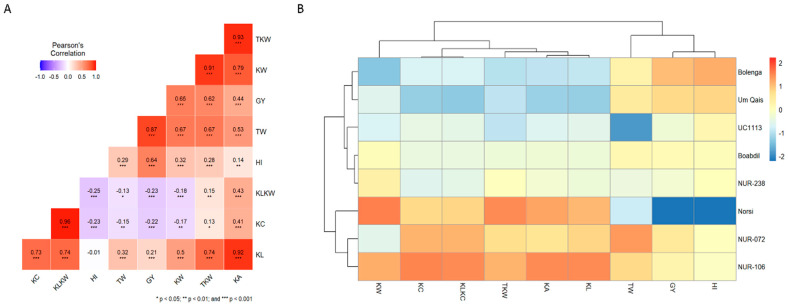
(**A**) Pairwise correlation (Pearson’s coefficients) using phenotypic data ((grain yield (GY), total weight (TW), harvest index (HI), thousand kernel weight (TKW), kernel area (KA), kernel width (KW), kernel length (KL), kernel circularity (KC) and kernel length to kernel width ration (KL:KW)) of eight durum wheat genotypes grown across 15 different environments. (**B**) Heatmap clustering using the phenotypic data of eight durum wheat genotypes grown across 15 different environments. The intensity of color in each figure corresponds to the value of each estimate.

Pairwise correlation based on Pearson’s coefficients was employed to examine the correlations between different traits (Figure 4A). Significant positive correlations (*p* ≤ 0.01) were found between TKW and TW, GY, HI as well as all kernel-related traits except for KC and KL:KW. Notably KC and KL:KW showed a strong positive correlation between each other. Conversely, KC and KL:KW were found to have negative correlations (*p* ≤ 0.01) with TW, GY, HI, and KW (Figure 4A). Using Heatmap cluster analysis for the studied traits, two distinct clusters were observed. The first cluster comprised the kernel-related traits with a clear clustering between KC and KL:KW as well as among TKW, KL, and KA. The second cluster consisted of GY, TW, and HI (Figure 4B). Furthermore, the genotypes dendogram identified three distinct clusters with Norsi, NUR-072, and NUR-106 formed one together, Bolenga and Um Qais grouped together in a second cluster and NUR-238, UC1113 *Yr36+Gpc-B1* and Boabdil were grouped in a single cluster (Figure 4B). Interestingly, the Norsi, NUR-072, and NUR-106 cluster exhibited higher values for all kernel-related traits except for NUR-072 and KW. The Bolenga and Um Qais cluster displayed higher values for GY, TW, and HI compared to other tested genotypes (Figure 4B).

### 2.3. Genotypes Performance and Stability Analysis

**Figure 5:**
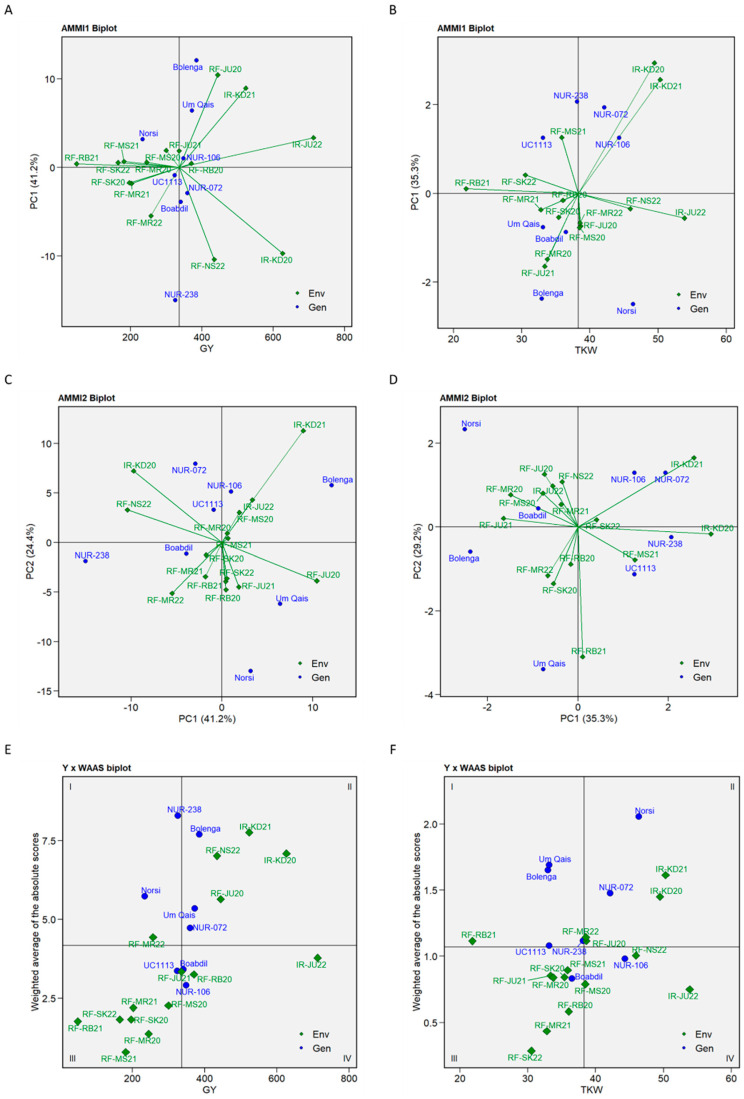
AMMI1 biplots for grain yield (**A**) and thousand kernel weight (**B**) using the means of eight durum wheat genotypes and environments against their respective IPC1 scores; AMMI2 biplots for grain yield (**C**) and thousand kernel weight (**D**) using their respective IPC1 and IPC2 scores; Means × WAAS biplots for grain yield (**E**) and thousand kernel weight (**F**) using the means of eight durum wheat genotypes and environments against their WAAS scores.

The AMMI analysis was carried out to identify the most stable and high-yielding genotypes. The AMMI analysis revealed significant effects of genotype, environment, and G × E interaction for both the GY and TKW traits, with the environmental variance being the largest contributor (Table S4). The AMMI analysis of kernel-related traits revealed substantial variation due to genotype effect for KA, KL, KC, and KL:KW, while KW variation was primarily attributed to environmental variance (Table S4). Regarding GY, the IPCA1 and IPCA2 explained 41.2% and 24.4% of variance attributed to the G × E interaction, respectively. For TKW, IPCA1 and IPCA2 explained 35.3% and 29.2% of the variance attributed to the G × E interaction, respectively. The AMMI1 GY biplot (IPC1 vs. GY) identified RF-JU20 (IPC1 = 10.45), RF-NS22 (IPC1 = −10.40), IR-KD20 (IPC1 = −9.72) and IR-KD21 (IPC1 = 8.96) as the most interactive environments. Bolenga exhibited the highest GY mean (385.42 g.m^−2^), followed by Um-Qais (372.55 g.m^−2^), NUR-072 (359.35 g.m^−2^) and NUR-106 (348.56 g.m^−2^) (Figure 5A). In addition, The AMMI1 GY biplot identified NUR-106 (IPC1 = 1.02) as the most stable among the high-yielding genotypes, demonstrating good performance and adaption to different environments. For TKW, the AMMI1 biplot identified IR-KD20 (IPC1 = 2.94) and IR-KD21 (IPC1 = 2.56) as the most interactive environments. Norsi, NUR-106 and NUR-072 were found to be superior genotypes producing TKW means above the average. Interestingly, NUR-106 and NUR-072 performed well in saline environments (IR-KD20 and IR-KD21), while Norsi was considered the least stable genotype among the genotypes with high TKW (Figure 5B).

The relationship between IPCA1 and IPCA2 scores was further investigated in the AMMI2 biplot analysis for GY and TKW and the results confirmed that most of interactive environments identified in AMMI1 were also suitable for discriminating tested genotypes for both traits (Figure 5C,D). Furthermore, Boabdil was identified as the most stable genotype for both traits, while Bolenga, Norsi, and NUR-238 were identified as the lowest stable genotypes for GY. For TKW, Um Qais, Bolenga, and Norsi were identified as the least stable genotypes. Interestingly, NUR-072 and NUR-106 exhibited adaptability to irrigated saline environments (IR-KD20 and IR-KD21) for both tested traits (Figure 5C,D).

Based on the IPCs of G × E interaction from the AMMI ANOVA and using the means of GY and TKW, WAASi biplots were generated to identify the best performance and stable genotypes (Figure 5E,F). For GY, Norsi and NUR-238 were identified as unstable genotypes in the first quadrant (I), whereas Bolenga, Um Qais and NUR-072 were found in the second quadrant (II) indicating they are highly productive but still considered unstable genotypes (Figure 5E). In the third quadrant (III), UC1113 *Yr36+Gpc-B1* was identified as a low-yielding and stable genotype, while in the fourth quadrant (VI), NUR-106 and Boabdil were identified as stable and productive genotypes. Regarding TKW, Bolenga, Um Qais, UC1113 *Yr36+Gpc-B1* and NUR-238 were identified in quadrant (I), Norsi and NUR-072 in quadrant (II), Boabdil in quadrant (III) and NUR-106 in quadrant (VI) indicating high stability and TKW mean value (Figure 5F). In addition, the FAI-BLUP, MTSI, WAASB and WAASBY indices identified NUR-106 among the most superior and stable genotypes (Figure S3).

**Figure 6:**
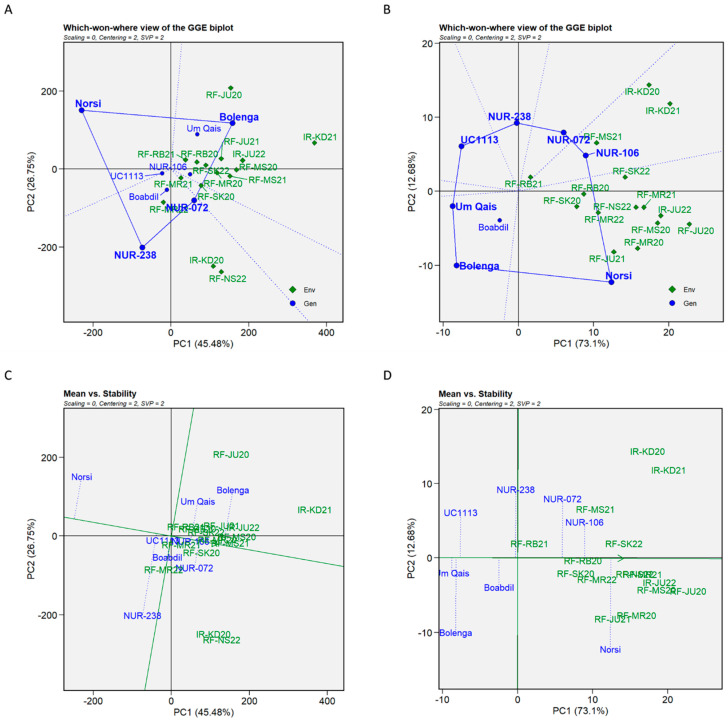
GGE biplot analysis of grain yield and thousand kernel weight for eight durum wheat genotypes tested across 15 environments. (**A**) The “which won and where” pattern for grain yield; (**B**) the “which won and where” pattern for thousand kernel weight; (**C**) stability analysis (the ‘mean vs. stability’ pattern) for grain yield; (**D**) stability analysis (the ‘mean vs. stability’ pattern) for thousand kernel weight.

The GGE biplot analysis was performed to assess genotype performance and specific adaptation to the tested environments. This analysis allowed the examination of the main effects of genotypes along with G × E interactions (GGE) using a two dimensional approximation with PC1 and PC2 values in order to identify the high-yielding ability and stability of tested genotypes. The GGE biplot analysis for ‘which-won-where’ and “mean vs. stability” patterns for the GW and TKW traits are presented in Figure 6. By considering the means across all environments and including all tested genotypes, PC1 and PC2 accounted for 72.23% and 85.78% of the variations in genotypes plus G × E interactions for the GW and TKW traits, respectively. Analyzing the ‘which-won-where’ patterns, the generated GGE biplots were divided into four sectors for GY and seven sectors for TKW (Figure 6A,B). For GY, the GGE biplot indicated that Bolenga was the vertex cultivar in most of the test environments (10 out of 15 environments) signifying its superior performance in these environments (Figure 6A). The same sector included Um Qais and NUR-106 genotypes, suggesting that both can perform well in the same environments where Bolenga wins. On the other hand, Norsi displayed poor performance across all testing environments and was considered the least productive compared to other tested genotypes. The GGE biplot analysis for TKW revealed that Norsi was the vertex cultivar in most of the tested environments (11 out of 15 environments) indicating its excellent performance and high TKW there (Figure 6B). In another sector, NUR-106 demonstrated good performance and high TKW in three environments including IR-KD20 and IR-KD21. In contrast, the vertex genotype Bolenga, Um Qais, UC1113 *Yr36+Gpc-B1*, NUR-238, and the non-vertex Boabdil did not win in any of the testing environments (Figure 6B).

For mean performance and stability (‘means vs. stability’) analysis, the averages of tested genotypes were ranked in reference to the average of the tested environments for GY and TKW (Figure 6C,D). Bolenga had the highest GY average (the most yielding genotype) followed by NUR-072, Um-Qais, NUR-106, Boabdil, UC1113 *Yr36+Gpc-B1*, NUR-238 and Norsi, which had the lowest GY average (Figure 6C). Furthermore, NUR-238, Bolenga, Norsi, and Um Qais were considered the least stable genotypes, while NUR-106 was the most stable genotype followed by UC1113 *Yr36+Gpc-B1*. For TKW, Norsi had the highest average (the heaviest kernels) followed by NUR-0106, NUR-072, NUR-238, Boabdil, UC1113 *Yr36+Gpc-B1*, Bolenga and Um Qais, which had the lowest grain yield average (Figure 6D). Um Qais was the most stable genotype followed by Boabdil and NUR-0106, while Norsi was the least stable genotype.

**Figure 7:**
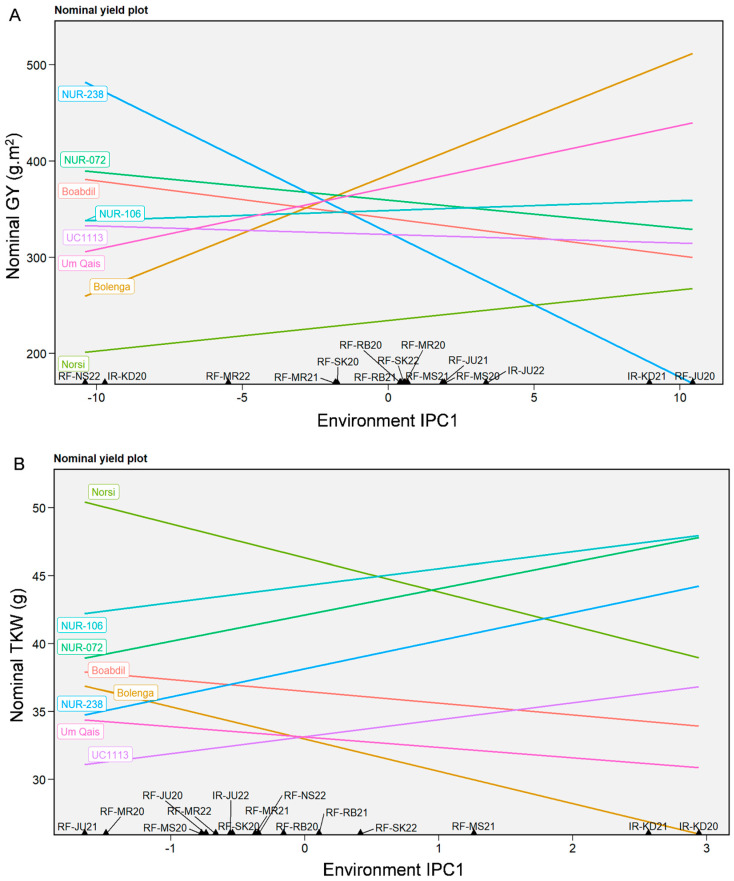
Nominal analysis for grain yield (GY) (**A**) and thousand kernel weight (TKW) (**B**) of eight durum genotypes as a function of the IPC1 scores of 15 tested environments.

Nominal analysis was conducted for GY, TKW, and kernel-related traits using AMMI IPC1 of each environment to interpret the “which-won-where” pattern and to identify genotypes that perform best at specific environments and as universal stable across environments (Figure 7). Regarding GY, UC1113 *Yr36+Gpc-B1* followed NUR-106 were identified as universal stable genotypes across tested environments (Figure 7A). For TKW, Um Qais emerged as a universal stable genotype across tested environments, whereas Norsi was clearly the most unstable genotype (Figure 7B). In the “which-won-where” pattern for GY, Bolenga followed by Um Qais emerged as winners in ten environments, while Norsi consistently displayed low yields across all tested environments (Figure 7A). For TKW, Norsi, NUR-106, and NUR-072 outperformed other tested genotypes, with Norsi winning in 12 environments (Figure 7B). Interestingly, Norsi did not perform well in IR-KD20 and IR-KD21 for TKW, while both NUR-106 and NUR-072 performed better suggesting that these two environments represent separate recommendation domains for TKW. The nominal analysis of kernel-related traits indicated that NUR-106 consistently performed as the winner for KA, KL, KC and KL:KW across most of tested environments, while significant variation was observed for KW with NUR-106 identified as the most stable genotype (Figure S4).

## 3. Discussion

The identification of high-yielding and stable genotypes for GY, TKW, and kernel-related traits is considered important for breeding new durum wheat varieties suitable for dry environments [25,26]. In this study, the effects of genotype, environment and G × E interaction were investigated on eight durum wheat genotypes with different kernel characteristics. These genotypes were analyzed for their agronomic performance and stability across different environments in Jordan. The analysis of stability revealed that the environment significantly contributed to the variation in GY in durum wheat cultivated in dry environments and this is consistent with the previous studies [27,28].

Norsi, a Jordanian landrace used in this study, was collected previously from a farmer field in Fakreh village (Ajloun Governorate-Jordan), is known for its high rainfall and temperate climatic conditions [29]. Norsi exhibited low GY and was the least stable among tested genotypes, which was expected considering that high-yielding varieties are typically bred to produce high GY and HI with better adaptability and stability across different environments compared to local landraces [30,31]. On the other hand, Norsi showed favorable performance for TKW and kernel-related traits compared to the high-yielding varieties such as Bolenga and Um Qais. It is worth noting that Norsi displayed great instability and performed poorly under saline environments (IR-KD20 and IR-KD-21). Previous studies have reported lower TKW in durum wheat landraces from eastern Mediterranean countries compared to western Mediterranean countries [32,33]. Additionally, Dencic et al. [34] reported lower GY and higher TKW mean values in landraces compared to improved varieties under drought conditions. In this study, Norsi exhibited unique characteristics as a landrace from eastern Mediterranean, particularly in terms of kernel-related traits that could be exploited to improve TKW in durum wheat under drought conditions.

In this study, Um Qais and Bolenga were identified as high-yielding genotypes across different environments, although their stability varied. Um Qais was assigned to sub-population 5 (ICARDA continental-dryland) based on the genetic diversity among 191 durum wheat accessions assigned, while Bolenga was not assigned to any specific sub-population suggesting different origins for both genotypes [35]. Interestingly, both genotypes exhibited smaller TKW and lower mean values for kernel-related traits, indicating that other yield components-related traits contribute to their high GY. Um Qais (also known as Cham-5 or ‘Om Rabi”) is a drought-tolerant cultivar derived from a cross between the Jordanian landrace ‘Hourani’ and Jori-c69, a high-yielding CIMMYT line [36]. It has been adopted by several countries in the southern Mediterranean region. Hourani is recognized for its excellent drought tolerance and high stability in eastern Mediterranean environments. It is characterized by its dense spikes, high number of kernels per spike, small kernel size, and round kernel shape resulting in low TKW values [37]. Lines derived from Hourani, including Um Qais, exhibit a high number of grains per spike and low TKW values, indicating a different strategy for increasing GY that is not related to TKW [38]. Um Qais has also been found to be high-yielding with relative stability under saline conditions [39], although some reports suggest it is moderately salt-tolerant [40]. However, AMMI analysis conducted on ICARDA durum wheat elites across 18 countries indicated that Um Qais was among the least stable genotype [41], which could explain the observed relative instability in this study. Bolenga is a Spanish cultivar that has been described previously as salt sensitive and susceptible to leaf rust [42,43]. It was selected from the ICARDA global panel of durum wheat [36] based on promising GY observed in preliminary field trials under drought conditions [44]. Bolenga performed exceptionally under different conditions in Jordan and was considered a winning genotype in 10 out of 15 environments. Similar to Um Qais, Bolenga exhibited low TKW values, and similar to Norsi, was considered one of the least stable genotypes for TKW. In a study by [45], different stability parameters failed to identify the most stable cultivar for TKW among local adapted varieties under semi-arid conditions. Modern durum wheat cultivars have been bred primarily for high GY potential without considering the stability of TKW in dry and hot environments [46]. Therefore, there is a need to identify and develop durum wheat genotypes that exhibit both good performance and stability for TKW under such conditions.

In this study, two RILs, namely NUR-072 and NUR-106, derived from a cross between Norsi and UC1113 *Yr36+Gpc-B1*, exhibited promising performance and were relatively stable for GY, TKW and other kernel-related characteristics. UC1113 *Yr36+Gpc-B1*, known for its stripe rust resistance, high protein content, and good yield potential and stability [47] performed well compared to Norsi, which showed poor performance for GY in this study. NUR-106, in particular, exhibited the highest stability for GY and produced a higher mean value than both parental lines. Interestingly, this line showed the highest stability in different tests for GY and TKW and it produced the highest mean values for different kernel-traits, particularly, KL and KC. NUR-106 was selected as the most stable genotype in this study using stability indices that relied on multi-traits analysis (MTSI and FAI-BLUP). Furthermore, NUR-072 and NUR-106 were found to perform well and similar to UC1113 *Yr36+Gpc-B1* for GY and TKW under rainfed conditions with supplementary irrigation (IR-JU22, IR-KD20 and IR-KD21). Additionally, under rainfed conditions with a sufficient amount of rainfall (not severe drought), both RILs surpassed UC1113 *Yr36+Gpc-B1* in terms of GY and TKW. For TKW, the performance of both RILs was superior to that of Norsi in IR-KD20 and IR-KD-21, suggesting that Norsi may have been affected by salinity. Interestingly, UC1113 *Yr36+Gpc-B1* exhibited its highest TKW values under salinity compared to other tested environments indicating its general adaptability to saline conditions. In terms of GY and TKW, improved wheat cultivars carrying *Rht-1b* are known to respond positively under high-input environments, suggesting their suitability for such conditions [31]. Drought and salinity are known to significantly affect TKW in durum wheat [48], but still, NUR-106 exhibited excellent performance and stability under dry and saline conditions, particularly for TKW and kernel-related traits making it a promising candidate for future improvement of kernel-related traits in durum wheat.

In this study, the variations observed in tested environments and genotypes emphasized the importance of TKW and associated kernel traits in breeding new lines with improved stability and their association with improved GY. Correlation analysis revealed a positive correlation between KL, KW, and TKW, which is in general agreement with previous studies [49,50]. High *H*^2^ values were also observed for kernel-related traits, which is consistent with previous studies [21,51]. Interestingly, KW exhibited lower *H*^2^ values compared to KL and this pattern aligns well with previous studies [52,53,54]. These findings highlight the importance of KL as a targeted trait in future durum wheat breeding programs. In this perspective, KL has been found important for test weight stability in selected winter and facultative wheat genotypes as well as for their quality and milling characteristics [18]. In fact, several stable QTLs were identified for KL in wheat material tested across different environments indicating that it was less affected by environmental factors and its suitability for genetic improvement in wheat [18,50]. The reported RILs with improved kernel characteristics in this study hold great potential for future research to understand the genetic basis of their stability under contrasting environments. Additionally, these RILs can be effectively utilized in durum wheat breeding programs to produce high-yielding varieties with enhanced kernel characteristics.

## 4. Materials and Methods

### 4.1. Plant Material

In this study, eight durum wheat genotypes were selected. These included Um Qais (synonymous with Omrabi5 (Jori-C69/Hourani), a Jordanian registered cultivar developed by ICARDA; Norsi, a durum wheat landrace collected in Jordan that was chosen for its high TKW and unique kernel-related characteristics [29]; two Spanish elite cultivars (entry 335 (Boabdil) and entry 336 (Bolenga) were selected from the ICARDA global panel of durum [36,44]; UC1113 *Yr36+Gpc-B1*, a high-yielding line carrying the *Yr36* and *Gpc*-*B1* alleles, was also included. This line was developed at UC-Davis (Davis, CA, USA) and obtained from the USDA-ARS national small grains collection (PI 638741); Finally, three durum wheat recombinant inbred lines (RILs) were selected from a F7-RIL population derived from a cross between Norsi and UC1113 *Yr36+Gpc-B1*. The three selected RILs, namely NUR-072; NUR-106 and NUR-238, are dwarf lines carrying *Rht-B1* and they were specifically selected for their high-yielding potential and special kernel-related characteristics, particularly high TKW.

### 4.2. Field Experiments

The selected durum wheat genotypes were cultivated over three growing seasons (2019–2020, 2020–2021, and 2021–2022) at various locations across Jordan. The field trials were carried out at the following research stations: Jubeiha agricultural research station (JU), representing a semi-humid environment and it is located in the University of Jordan/Amman governorate (32°00′40″ N, 35°52′24″ E; elevation: 990 m) with an average annual precipitation of 520 mm and it has a “clay loam” soil type; Al-Khaldieh station for saline research (KD; NARC-Jordan), representing a saline environment and it is located in Mafraq governorate (32°09′56″ N, 36°17′24″ E; elevation: 628 m) with an average annual precipitation of 150 mm and it has a “clay loam” soil type with a salinity level of 9.44 ds/m; Maru agricultural research station (MR; NARC-Jordan), representing a semi-arid environment with high disease pressure (particularly leaf and stripe rusts) and it is located in Irbid governorate (32°36′26″ N, 35°54′04″ E; elevation: 525 m) with an average annual precipitation of 400 mm and it has a “clay” soil type; Mushagar agricultural research station (MS; NARC-Jordan), representing a semi-arid environment and it is located in Madaba governorate (31°46′27″ N, 35°48′00″ E; elevation: 791 m) with an average annual precipitation of 330 mm and it has a “clay” soil type; Rabbah agricultural research station (RB; NARC-Jordan) representing a semi-arid environment with frequent drought conditions and it is located in Al-Karak governorate (31°16′31″ N, 35°44′46″ E; elevation: 925 m) with an average annual precipitation of 320 mm and it has a “clay loam” soil type; Al-Shoubak College research station (SK; Balaqa Applied University) representing an arid environment with frequent frost conditions and it is located in Ma’an governorate (30°29′39″ N, 35°31′38″ E; elevation: 1450 m) with an average annual precipitation of 222 mm and it has a “clay loam” soil type. Al-Nasareyeh agriculture research station represents a semi-arid environment with frequent heat conditions and it is located in Nablus governorate (32°15′39.6″ N, 35°21′38.2″ E; elevation: 120 m) with an average annual precipitation of 300 mm and it has a “loam” soil type. For the purpose of data analysis, each combination of location and year was treated as an independent environment.

All field trials were sown during the last week of December in each growing season and harvested when the entire plot reached the harvest maturity stage (Zadoks scale: GS87) [55]. The tested genotypes were cultivated under rainfed conditions without any supplementary irrigation except in KD where plots were supplementary irrigated from a saline water source using a drip irrigation system employed following the standard stations programs. In JU during the 2022 season, the plots were irrigated from a non-saline water source using a drip irrigation system employed following the standard stations programs. In addition, supplementary irrigation (equivalent to 100 mm of rainfall) was applied once at SK in April 2022 to avoid crop failure due to severe drought conditions. Seeding rates were adjusted based on germination tests to obtain a plant density of 150 plants.m^−2^. The field trials followed a fallow in the crop rotation and were established in a block area that consisting of eight plots. Each plot represented a tested genotype and three blocks (replicates) were used for each field trial. The plots were randomly distributed within the block, with each plot area equal to 4.5 m^2^ (1.5 m width × 3 m length). There were six rows in each plot with a row spacing of 25 cm and a plot to plot spacing of 75 cm. The experimental design employed a randomized complete block design (RCBD) with three replications. Seed sowing was done by hand broadcasting following conventional tillage using a chisel plough and rotavator. Experimental plots were managed following the standard agricultural practices at each location. Weather stations at the field sites recorded precipitation and temperatures during the growing seasons.

At the harvest maturity stage, plants were harvested and the following traits were recorded: GY (g.m^−2^) was measured as the total GY after all harvested spikes were separated and threshed and the grains were cleaned. Total weight (TW in g.m^−2^): dry weight of the whole harvested plants, including straw and spikes. Harvest index (%) calculated using the equation: (GY/TW) × 100. The harvested grains were analyzed for the following kernel characteristics using the MARVIN seed analyzer machine (Marvin-GTA, Sensorik GmbH, Version 5.0): TKW (in g), kernel area (KA: mm^2^). kernel width (KW: mm), kernel length (KL: mm), kernel circularity (KC: mm), and the KL:KW ratio.

### 4.3. Data Analysis

For statistical analysis, the multi-environment field trials data were analyzed using the linear mixed model analysis from the lme4 package in R embedded in the META-R software [56]. The analysis was carried out considering the RCBD design and using the restricted maximum likelihood (REML) method assuming fixed effects of environments, while all other terms were treated as random effects. Variance components and the significance level of genotype, environment and G × E interaction effects were determined and the best linear unbiased prediction (BLUP) for tested genotypes in each individual environment or combined across all environments were computed. In addition, the coefficients of variation (CV%), broad-sense heritability (*H*^2^), least significance difference (LSD, *p ≤ 0.05*) and the grand mean for each trait were computed. Pearson’s correlation coefficients were calculated in R using the corrplot package using the BLUPs of genotypes across tested environments. The pheatmap package v1.0.8 in R (https://cran.r-project.org/web/packages/pheatmap/index; accessed on 1 April 2022) was used to construct a heatmap and a hierarchical cluster using Euclidean distances and Ward.D2 method using the BLUPs of the genotypes across all tested environments.

The performance of tested genotypes across different environments and their stability for the GY, TKW and other selected traits was analyzed using various functions embedded in the metan package [57] in R. These functions included heatmap function for genotype’s performance across environments; the GGE biplot function [58], the additive main effects and multiplicative interactions (AMMI; [59]); the Olivoto’s multi-trait stability index (MTSI; [60]; the multi-trait index based on factor analysis and ideotype-design (FAI-BLUP; [61]); the weighted average of absolute scores (WAAS; [60]); the weighted average of absolute scores from BLUP interaction matrix (WAASB; [60]); the weighted average of stability (WAASB) and mean performance (Y) (WAASBY; [60]).

## 5. Conclusions

In conclusion, the obtained results revealed significant differences among environments, genotypes, and genotype-by-environment (G × E) interactions for studied traits and in particular kernel-related traits. In this study, NUR-106 emerged as the most stable genotype for several analyzed traits, holding promise for future studies to dissect the genetic basis underlying kernel-related characteristics and their relationship with other yield-components under drought and saline conditions. The findings of this study provide valuable insights for breeders seeking to develop high-yielding and stable durum wheat varieties with improved kernel characteristics, specifically suited for cultivation in dry areas.

## Data Availability

Data are not publicly available due to privacy or ethical restriction. The datasets supporting the results of this article will be freely available upon reasonable request from Yasser Bustanji and Ayed M. Al-Abdallat.

## Supplementary Materials

The following supporting information can be downloaded at: https://www.mdpi.com/article/10.3390/plants12142664/s1, Table S1. Rainfall amounts (mm on monthly basis) and average maximum and minimum temperatures (°C on monthly basis) in the tested environments. Table S2. Combined analysis of variance (ANOVA), grand mean values, broad-sense heritability (*H*^2^) and the coefficient of variation (CV) for nine traits collected from eight durum wheat genotypes tested across 15 different environments. Table S3. Individual environments and combined overall BLUPs values for nine traits recorded in eight durum wheat tested across 15 environments. Table S4. AMMI analysis of variance for GY, TKW and kernel-related traits in eight durum wheat genotypes tested across 15 different environments. Figure S1. Heatmaps for kernel-related traits (KA, KW, KL, KC and KL:KW) performance in eight durum wheat genotypes across 15 tested environments. Figure S2. Kernels of six selected durum wheat genotypes (Norsi, UC1113, NUR-072, NUR-106, Um Qais and Bolenga) grown under supplementary irrigated (IR-JU22) or rainfed (RF-NS22) conditions. Figure S3. FAI-BLUP (A), MTSI (B), WAASBY (C) and WAASB (D) indices for eight durum wheat genotypes tested across 15 environments. Figure S4. Nominal analysis for kernel-related traits (KA, KW, KL, KC and KLKW) of eight durum genotypes as a function of the IPC1 scores of 15 tested environments.
