# Supplementary material for: Performance and Stability Analysis of Selected Durum Wheat Genotypes Differing in Their Kernel Characteristics
Source: Plants (Basel). 2023 Jul 16;12(14):2664. doi: 10.3390/plants12142664 (PMC10384256; doi:10.3390/plants12142664)
Supplement: Supplementary file 1 [file plants-12-02664-s001.zip › Supplementary Figures S1-S4.pdf]

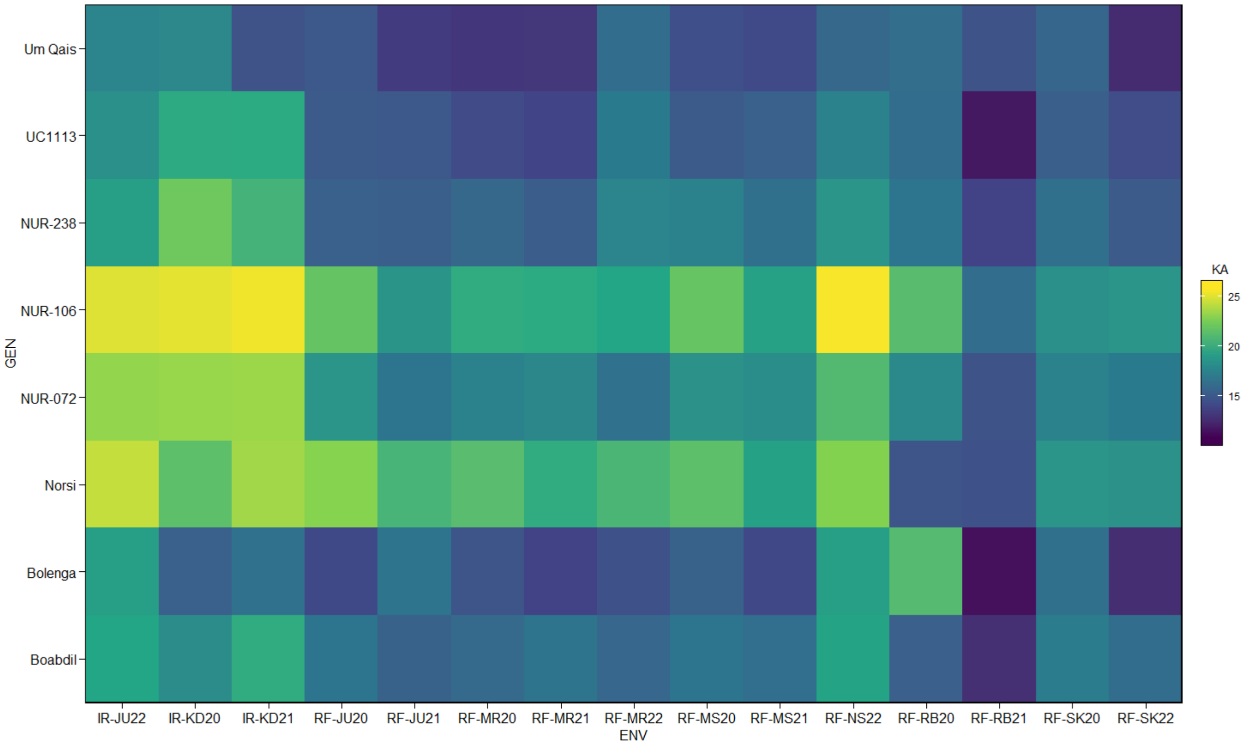

Um Qais
UC1113
NUR-238
NUR-106
NUR-072
Norsi
Bolenga
Boabdil
GEN
IR-JU22 IR-KD20 IR-KD21 RF-JU20 RF-JU21 RF-MR20 RF-MR21 RF-MR22 RF-MS20 RF-MS21 RF-NS22 RF-RB20 RF-RB21 RF-SK20 RF-SK22
ENV
KA
25
20
15


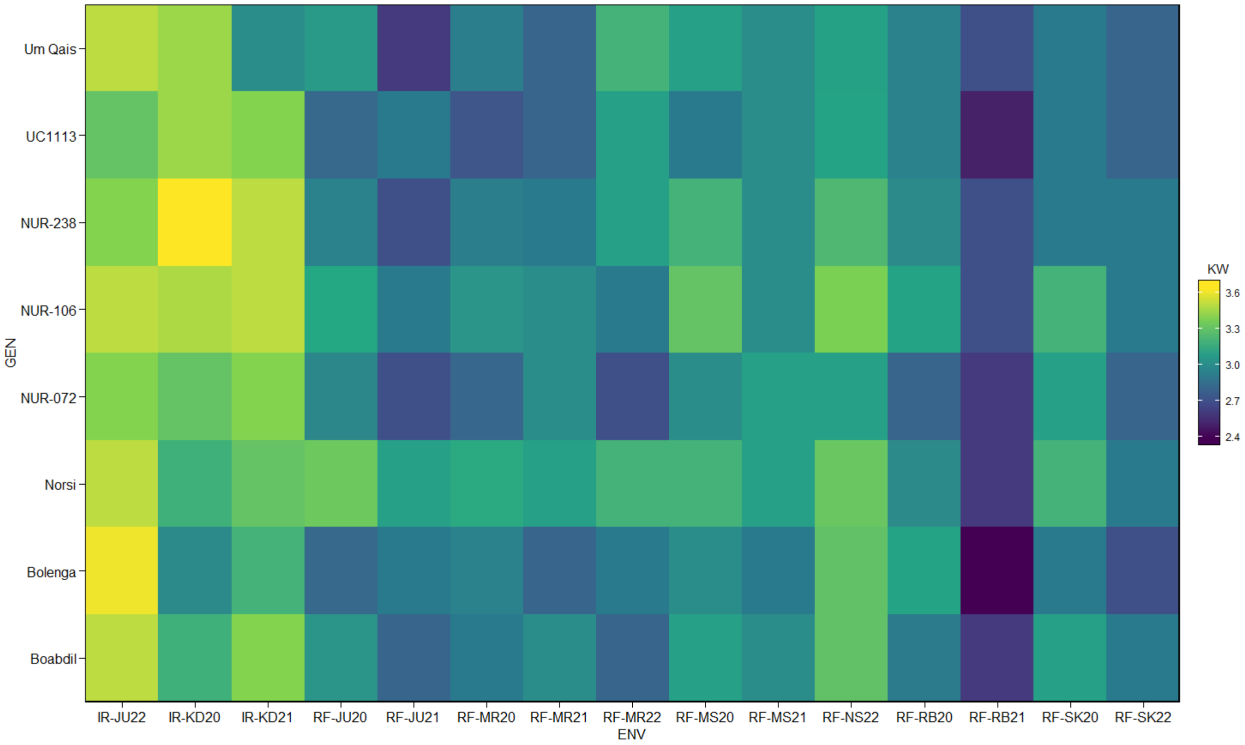

Um Qais
UC1113
NUR-238
NUR-106
NUR-072
Norsi
Bolenga
Boabdil
GEN
IR-JU22 IR-KD20 IR-KD21 RF-JU20 RF-JU21 RF-MR20 RF-MR21 RF-MR22 RF-MS20 RF-MS21 RF-NS22 RF-RB20 RF-RB21 RF-SK20 RF-SK22
ENV
KW
3.6
3.3
3.0
2.7
2.4

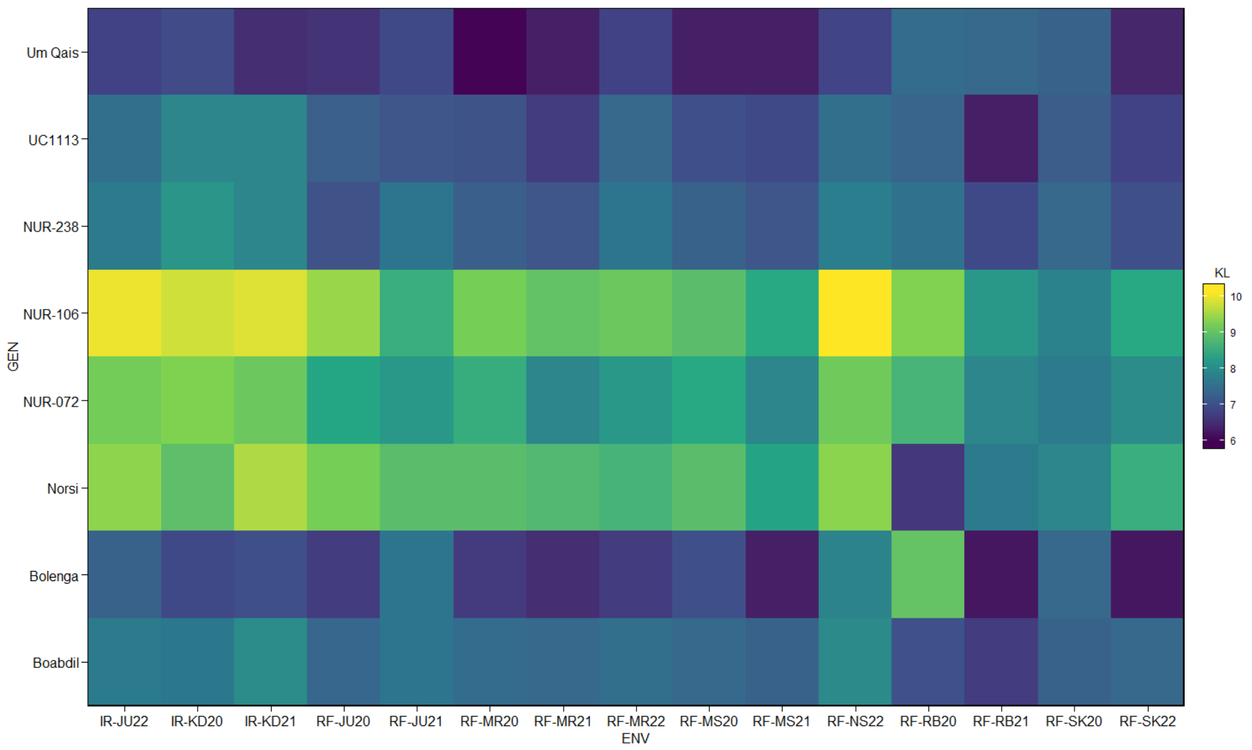

Um Qais
UC1113
NUR-238
NUR-106
NUR-072
Norsi
Bolenga
Boabdil
GEN
IR-JU22
IR-KD20
IR-KD21
RF-JU20
RF-JU21
RF-MR20
RF-MR21
RF-MR22
RF-MS20
RF-MS21
RF-NS22
RF-RB20
RF-RB21
RF-SK20
RF-SK22
ENV
KL
10
9
8
7
6


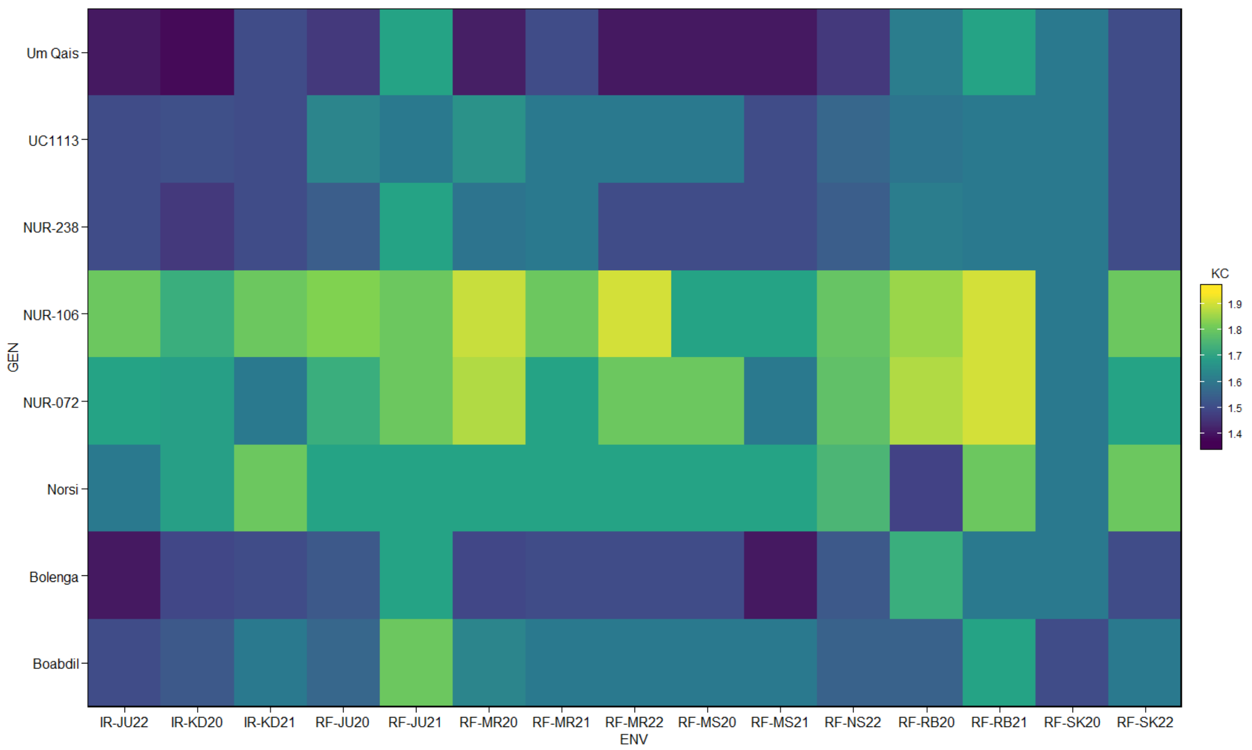

Um Qais
UC1113
NUR-238
NUR-106
NUR-072
Norsi
Bolenga
Boabdil
GEN
IR-JU22
IR-KD20
IR-KD21
RF-JU20
RF-JU21
RF-MR20
RF-MR21
RF-MR22
RF-MS20
RF-MS21
RF-NS22
RF-RB20
RF-RB21
RF-SK20
RF-SK22
ENV
KC
1.9
1.8
1.7
1.6
1.5
1.4

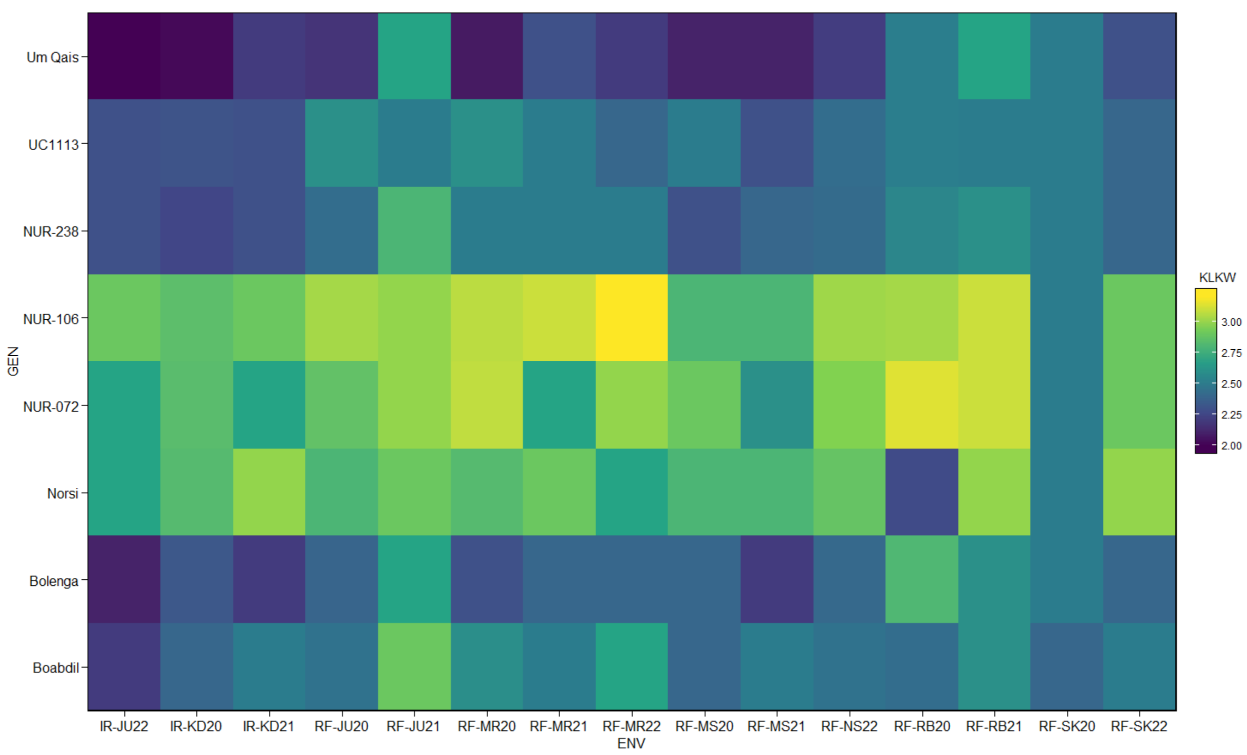


**Figure S1.** Heatmaps for kernel-related traits (KA, KW, KL, KC and KL:KW) performance in eight durum wheat genotypes across 15 tested environments.

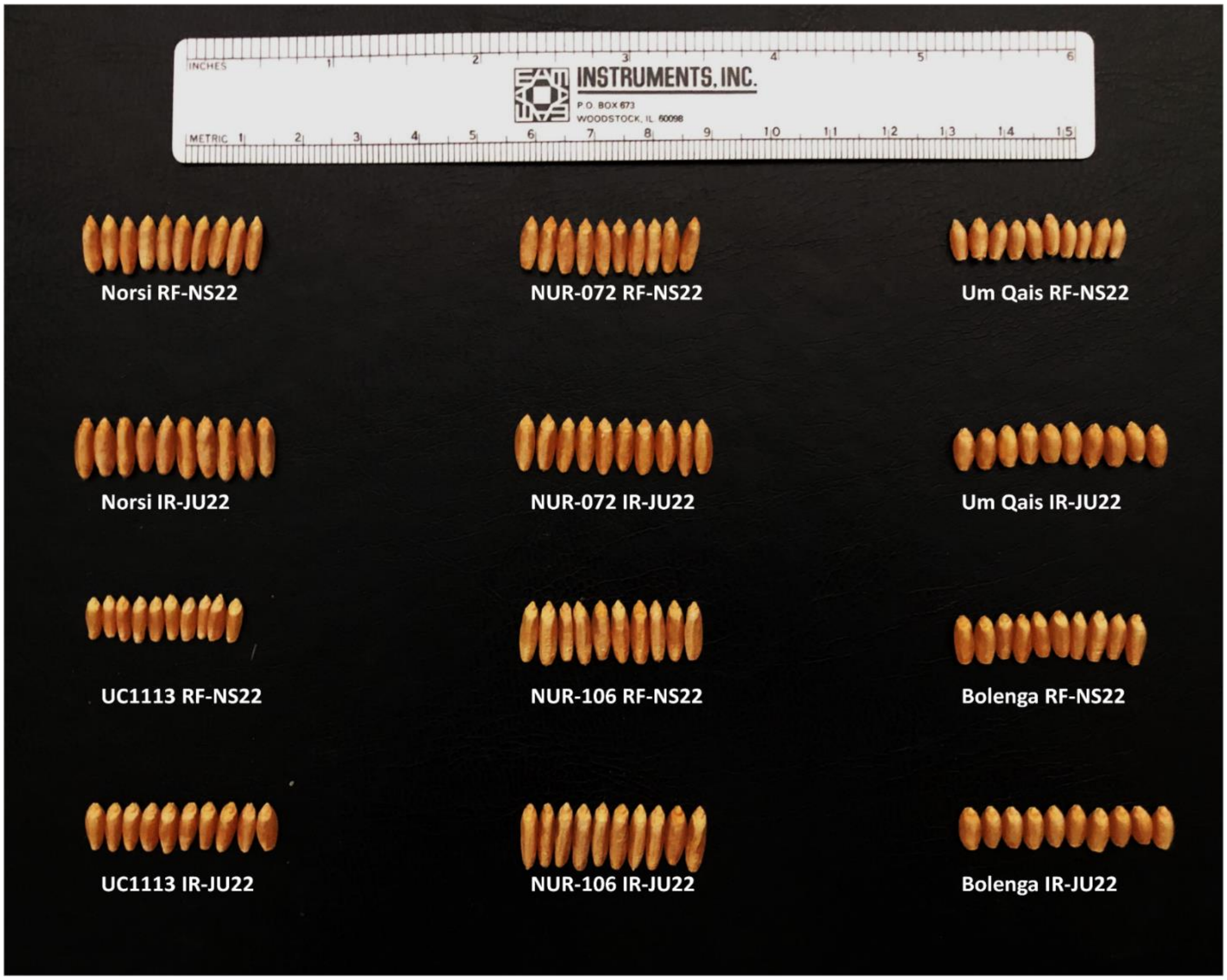


**Figure S2.** Kernels of six selected durum wheat genotypes (Norsi, UC1113, NUR-072, NUR-106, Um Qais and Bolenga) grown under supplementary irrigated (IR-JU22) or rainfed (RF-NS22) conditions.

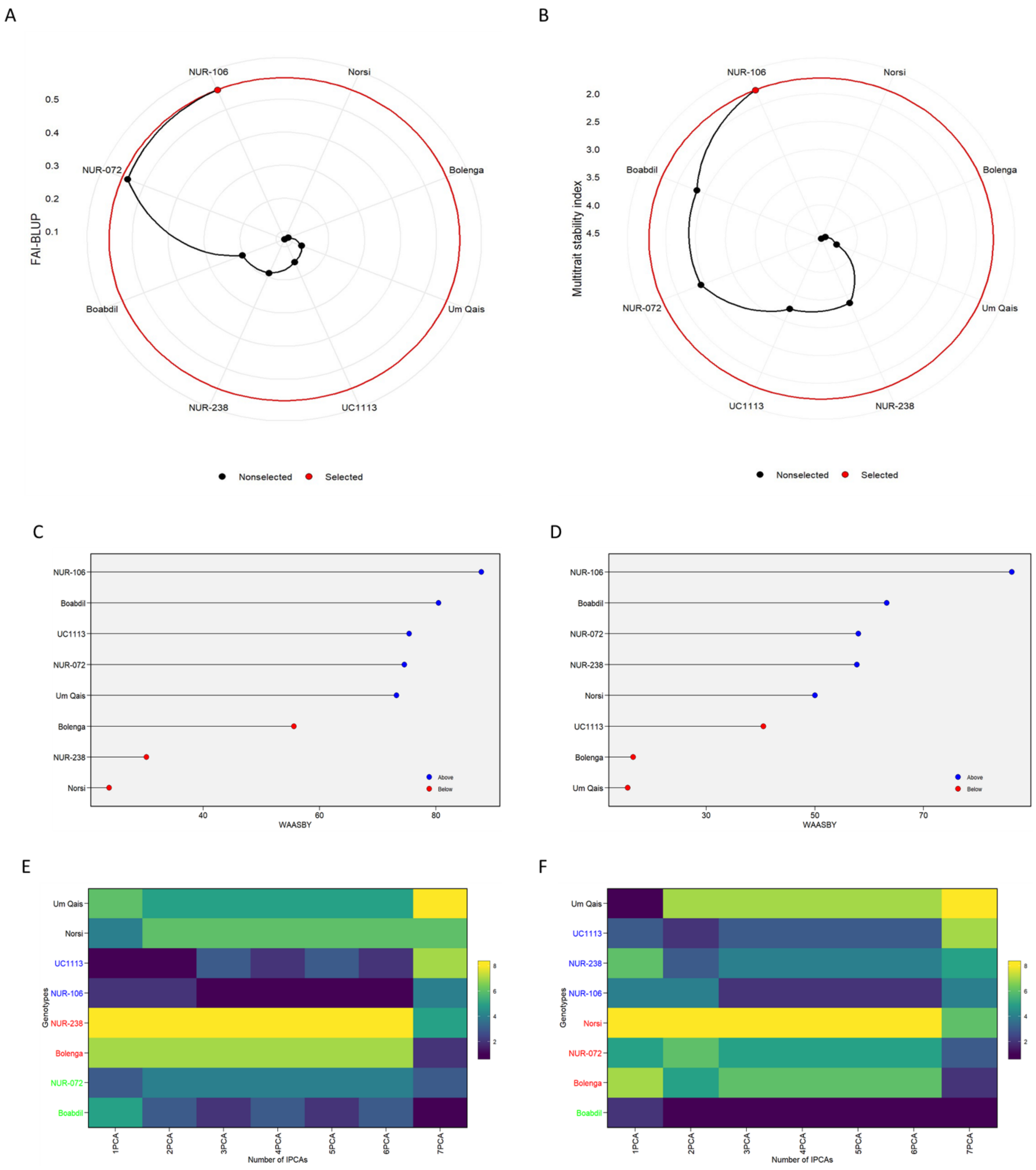


**Figure S3.** FAI-BLUP (A), MTSI (B), WAASBY (C) and WAASB (D) indices for eight durum wheat genotypes tested across 15 environments.

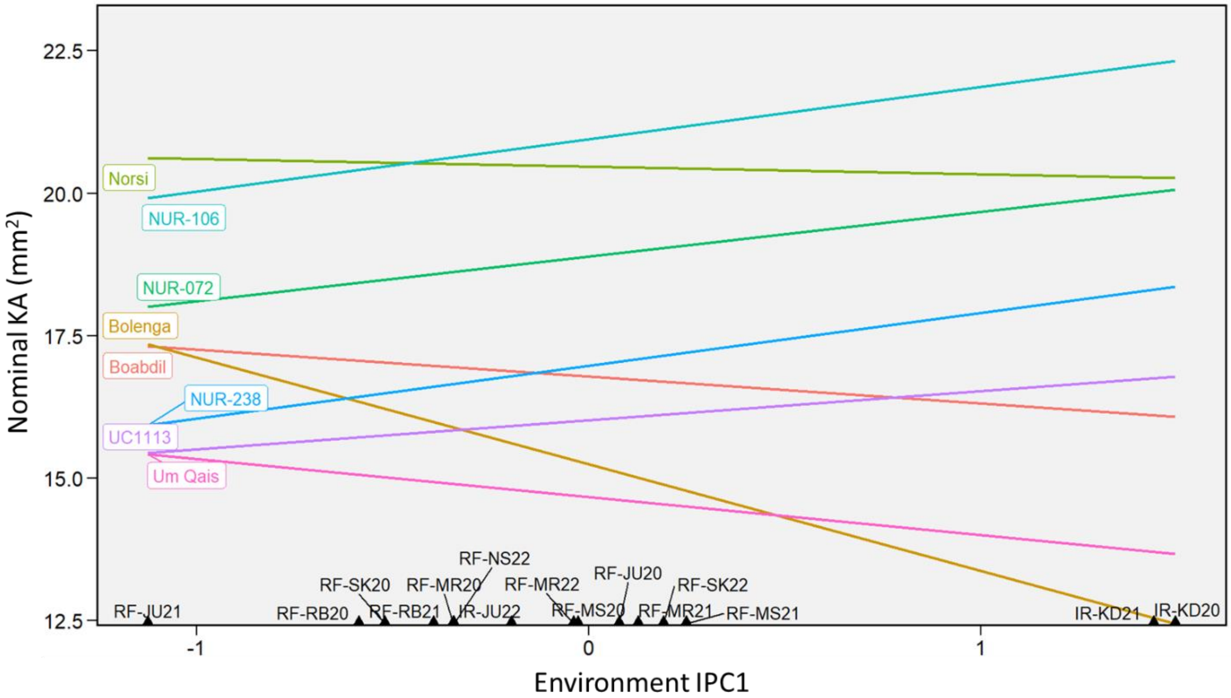

Nominal KA (mm²)
Environment IPC1
Norsi
NUR-106
NUR-072
Bolenga
Boabdil
NUR-238
UC1113
Um Qais
RF-JU21
RF-RB20
RF-SK20
RF-RB21
RF-MR20
RF-NS22
IR-JU22
RF-MR22
RF-MS20
RF-JU20
RF-MR21
RF-SK22
RF-MS21
IR-KD21
IR-KD20


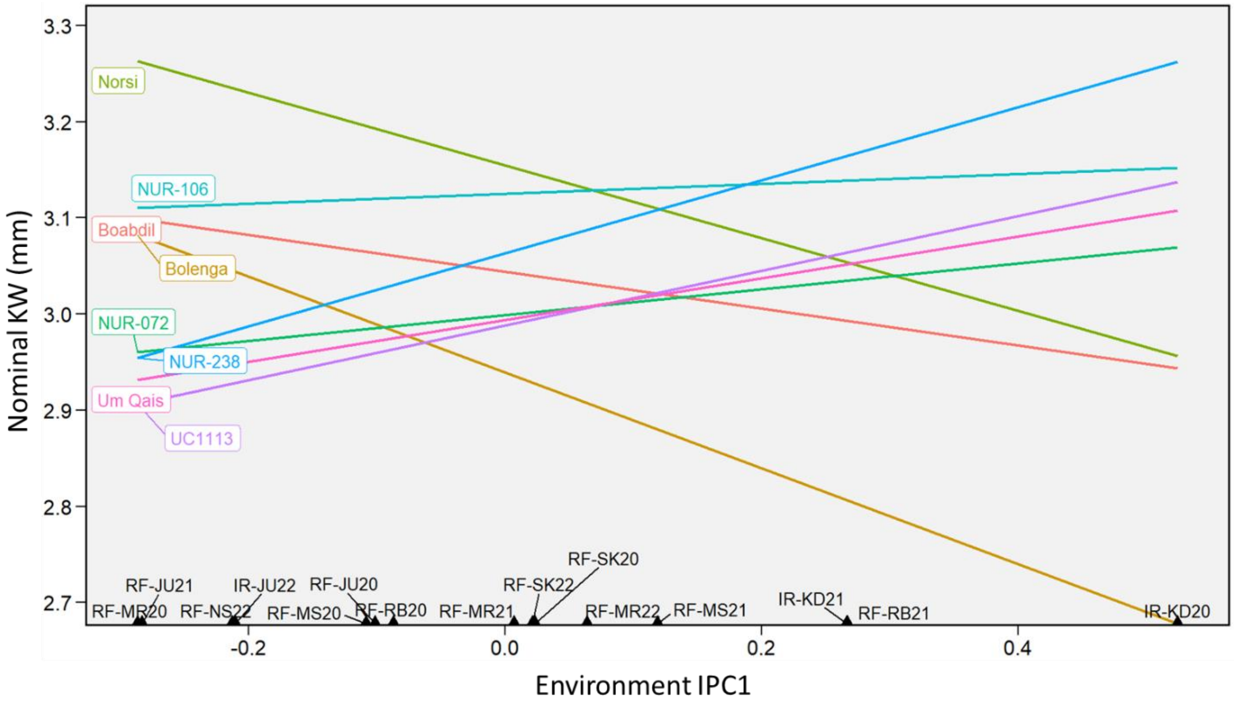

Nominal KW (mm)
Environment IPC1
Norsi
NUR-106
Boabdil
Bolenga
NUR-072
NUR-238
Um Qais
UC1113
RF-JU21
RF-MR20
RF-NS22
IR-JU22
RF-MS20
RF-JU20
RF-RB20
RF-MR21
RF-SK22
RF-SK20
RF-MR22
RF-MS21
IR-KD21
RF-RB21
IR-KD20

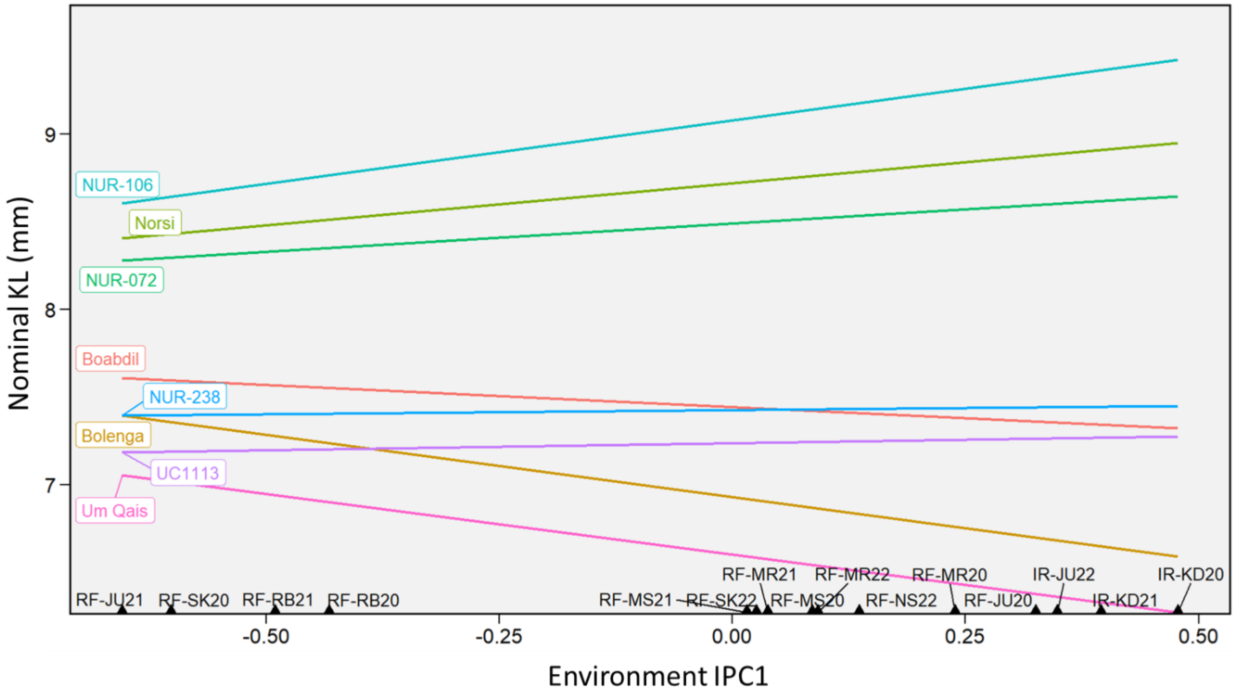
Nominal KL (mm)
Environment IPC1
NUR-106
Norsi
NUR-072
Boabdil
NUR-238
Bolenga
UC1113
Um Qais
RF-JU21
RF-SK20
RF-RB21
RF-RB20
RF-MS21
RF-SK22
RF-MR21
RF-MS20
RF-MR22
RF-NS22
RF-MR20
RF-JU20
IR-JU22
IR-KD21
IR-KD20

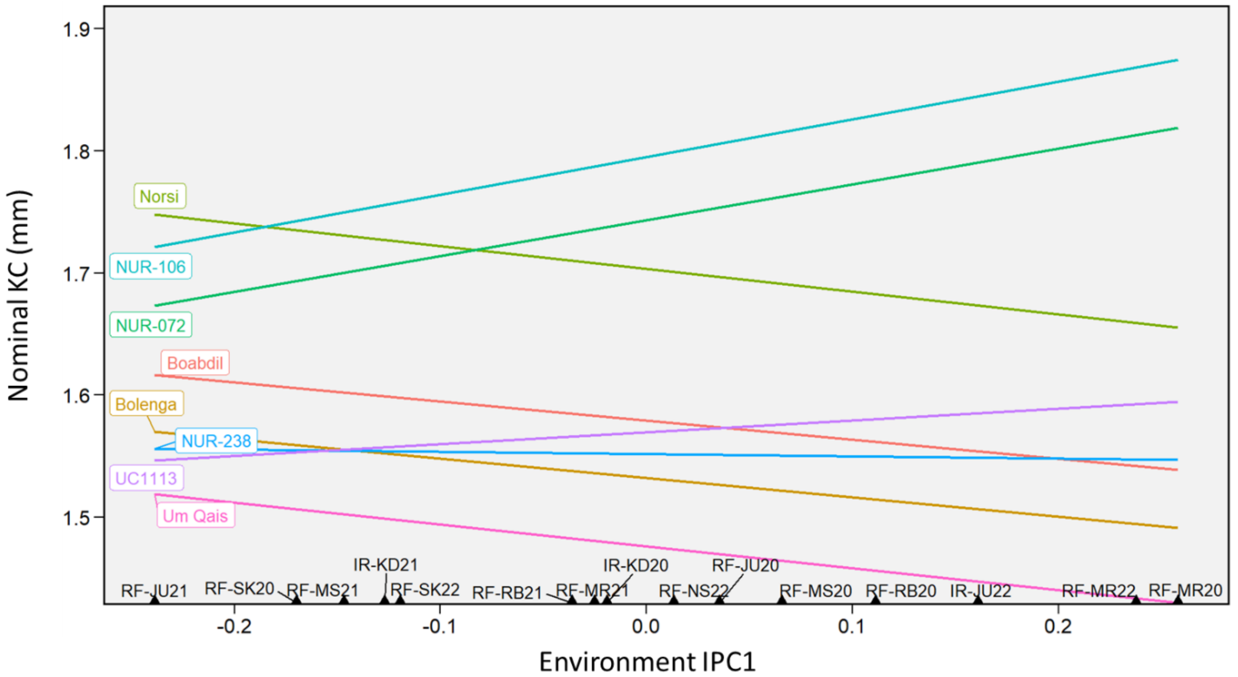
Nominal KC (mm)
Environment IPC1
Norsi
NUR-106
NUR-072
Boabdil
Bolenga
NUR-238
UC1113
Um Qais
RF-JU21
RF-SK20
RF-MS21
IR-KD21
RF-SK22
RF-RB21
RF-MR21
IR-KD20
RF-NS22
RF-JU20
RF-MS20
RF-RB20
IR-JU22
RF-MR22
RF-MR20

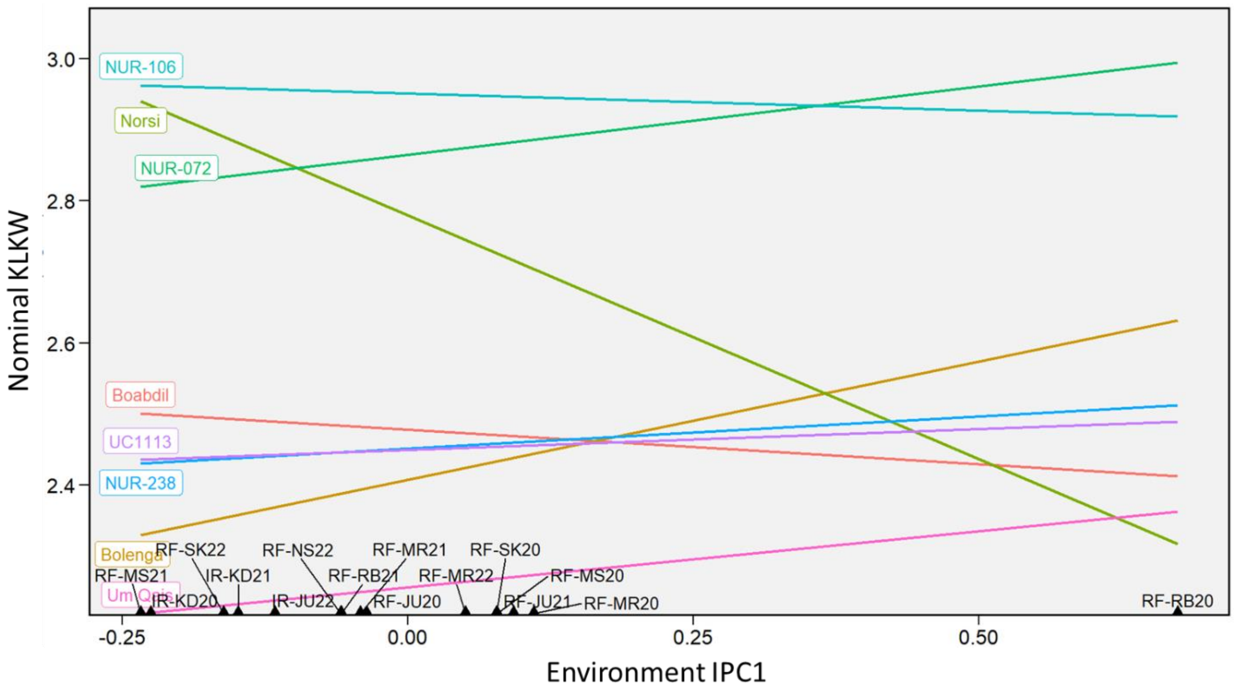


**Figure S4.** Nominal analysis for kernel-related traits (KA, KW, KL, KC and KL:KW) of eight durum genotypes as a function of the IPC1 scores of 15 tested environments.
