# Supplementary material for: Performance and Stability Analysis of Selected Durum Wheat Genotypes Differing in Their Kernel Characteristics
Source: Plants (Basel). 2023 Jul 16;12(14):2664. doi: 10.3390/plants12142664 (PMC10384256; doi:10.3390/plants12142664)
Supplement: Supplementary file 1 [file plants-12-02664-s001.zip › Supplementary Table S1.pdf]

Table S1. Rainfall amounts (mm on monthly basis) and average maximum and minimum temperatures (°C on monthly basis) in the tested environments.

Environment		November	December	January	February	March	April	May	June	Total
**RF-JU20**	**Maximum**	**21.91**	**15.12**	**10.91**	**13.48**	**17.85**	**21.88**	**29.21**	**30.76**	
	**Minimum**	**9.84**	**5.79**	**4.80**	**6.28**	**8.37**	**10.71**	**15.25**	**17.47**	
	**Rainfall**	**12.50**	**153.30**	**234.30**	**103.50**	**187.80**	**24.50**	**27.00**	**0.00**	**742.90**
**IR-KD20**	**Maximum**	**23.00**	**15.60**	**11.92**	**14.91**	**18.13**	**25.31**	**30.97**	**32.03**	
	**Minimum**	**9.10**	**4.00**	**3.00**	**3.94**	**6.74**	**8.94**	**12.66**	**13.86**	
	**Rainfall**	**12.90**	**44.50**	**39.90**	**42.40**	**66.50**	**15.80**	**7.50**	**0.00**	**229.50**
**RF-MR20**	**Maximum**	**26.10**	**17.30**	**12.20**	**14.40**	**19.90**	**24.30**	**31.30**	**31.10**	
	**Minimum**	**8.50**	**6.20**	**4.50**	**3.00**	**6.70**	**11.00**	**14.10**	**16.90**	
	**Rainfall**	**5.10**	**104.60**	**135.10**	**64.00**	**85.30**	**28.40**	**18.50**	**0.00**	**441.16**
**RF-MS20**	**Maximum**	**28.00**	**15.90**	**11.70**	**13.60**	**19.41**	**23.63**	**30.67**	**32.43**	
	**Minimum**	**2.00**	**5.50**	**3.20**	**4.60**	**6.09**	**7.06**	**12.12**	**13.26**	
	**Rainfall**	**21.70**	**66.70**	**152.50**	**84.80**	**111.80**	**3.80**	**13.10**	**0.00**	**454.40**
**RF-RB20**	**Maximum**	**22.00**	**14.80**	**10.50**	**12.50**	**17.30**	**21.70**	**29.30**	**30.42**	
	**Minimum**	**9.80**	**5.30**	**3.60**	**4.60**	**6.80**	**7.40**	**11.50**	**18.50**	
	**Rainfall**	**6.50**	**31.50**	**195.80**	**26.80**	**108.00**	**4.00**	**2.50**	**0.00**	**375.10**
**RF-SK20**	**Maximum**	**19.33**	**12.72**	**13.14**	**12.02**	**15.36**	**18.63**	**28.90**	**30.81**	
	**Minimum**	**4.21**	**2.85**	**-1.33**	**-1.10**	**0.52**	**4.51**	**9.81**	**10.64**	
	**Rainfall**	**0.20**	**19.10**	**1.40**	**95.80**	**112.50**	**2.00**	**10.80**	**0.00**	**241.80**
**RF-JU21**	**Maximum**	**20.87**	**18.74**	**16.00**	**17.05**	**15.84**	**25.01**	**30.55**	**29.37**	
	**Minimum**	**8.90**	**4.20**	**1.30**	**4.50**	**5.70**	**7.50**	**15.90**	**16.40**	
	**Rainfall**	**19.70**	**108.50**	**206.90**	**167.30**	**107.50**	**7.70**	**0.00**	**0.00**	**617.60**

Environment		November	December	January	February	March	April	May	June	Total
IR-KD21	Maximum	25.60	25.22	20.61	22.77	17.50	28.46	32.37	34.45	
	Minimum	6.41	1.89	-2.17	0.79	1.43	2.76	12.13	12.53	
	Rainfall	0.00	28.00	32.70	38.00	3.50	0.00	0.00	0.00	102.00
RF-MS21	Maximum	24.87	22.74	19.62	20.05	18.84	28.30	32.55	33.21	
	Minimum	10.05	6.48	1.31	4.46	5.73	7.49	15.87	16.36	
	Rainfall	41.90	15.00	119.50	68.00	47.30	0.00	0.00	0.00	291.70
RF-MR21	Maximum	22.50	18.10	16.50	17.60	16.90	26.40	31.30	30.70	
	Minimum	13.20	7.10	5.90	5.60	5.90	10.70	15.80	16.20	
	Rainfall	63.00	49.00	107.50	138.00	43.00	0.00	0.00	0.00	400.50
RF-RB21	Maximum	21.22	19.01	16.73	16.87	15.75	28.24	30.51	28.92	
	Minimum	11.60	7.71	2.69	4.59	7.23	8.23	17.39	18.12	
	Rainfall	39.00	20.50	66.80	48.50	17.40	0.00	0.00	0.00	192.20
IR-JU22	Maximum	27.40	20.10	10.50	12.50	12.80	26.60	27.20	30.12	
	Minimum	10.00	7.70	2.60	3.80	2.00	10.00	11.90	15.40	
	Rainfall	21.90	108.50	173.00	159.90	97.90	10.50	0.00	0.00	581.00
RF-NS22	Maximum	27.90	20.00	16.80	19.40	19.70	31.90	33.50	37.50	
	Minimum	15.20	9.60	5.30	7.40	7.00	13.80	16.60	20.60	
	Rainfall	18.70	68.60	89.50	51.50	71.00	5.50	1.00	0.00	305.80
RF-MR22	Maximum	26.60	20.60	16.60	19.20	19.90	29.60	31.50	33.10	
	Minimum	16.00	10.10	6.50	8.50	8.30	15.60	18.60	18.50	
	Rainfall	12.50	105.50	109.90	53.35	94.00	0.00	0.00	0.00	375.25
RF-SK22	Maximum	20.80	13.30	8.50	11.70	13.40	25.70	24.40	28.10	
	Minimum	3.30	-0.30	-2.70	-2.00	-1.50	5.50	7.90	11.10	
	Rainfall	4.40	8.80	68.20	24.60	7.60	100 (S.I)	0.00	0.00	213.60
