# Supplementary material for: Performance and Stability Analysis of Selected Durum Wheat Genotypes Differing in Their Kernel Characteristics
Source: Plants (Basel). 2023 Jul 16;12(14):2664. doi: 10.3390/plants12142664 (PMC10384256; doi:10.3390/plants12142664)
Supplement: Supplementary file 1 [file plants-12-02664-s001.zip › Supplementary Table S2.pdf]

Table S2. Combined analysis of variance (ANOVA), grand mean values, broad-sense heritability (*H^2*) and the coefficient of variation (CV) for nine traits collected from eight durum wheat genotypes tested across 15 different environments.

**Statistic**	**TW**	**GY**	**HI**	**TKW**	**KA**	**KW**	**KL**	**KC**	**KLKW**
**Genotype Variance**	1042.160	1825.018	11.175	27.383	5.441	0.005	0.811	0.012	0.062
**GenxLoc Variance**	24779.354	4491.951	16.424	15.880	1.157	0.010	0.061	0.001	0.007
**Residual Variance**	10731.418	1462.309	7.615	3.900	0.473	0.005	0.072	0.003	0.012
**Genotype Significance**	0.3973	9.11E-06	1.10E-08	1.27E-18	6.27E-33	3.59E-06	3.95E-48	1.38E-30	6.44E-39
**GenxEnv Significance**	1.14E-36	7.75E-45	1.04E-34	3.56E-58	1.68E-40	4.04E-34	1.70E-15	0.00182	1.01E-09
**Percentage of Variation-Genotype**	2.851	23.460	31.73467	58.014	76.897	23.591	85.923	76.283	76.914
**Percentage of Variation-GenxEnv**	67.790	57.74253	46.640	33.597	16.313	51.158	6.474	7.078	8.267
**Percentage of Variation-Residual**	29.359	18.798	21.626	8.389	6.790	25.251	7.603	16.639	14.819
**Grand Mean**	1078.929	336.274	29.462	38.316	17.502	3.038	7.741	1.629	2.580
**Heritability (H2)**	0.355	0.846	0.898	0.960	0.984	0.856	0.993	0.986	0.989
**CV (%)**	9.601	11.372	9.367	5.154	3.930	2.310	3.455	3.528	4.226
