# Supplementary material for: Performance and Stability Analysis of Selected Durum Wheat Genotypes Differing in Their Kernel Characteristics
Source: Plants (Basel). 2023 Jul 16;12(14):2664. doi: 10.3390/plants12142664 (PMC10384256; doi:10.3390/plants12142664)
Supplement: Supplementary file 1 [file plants-12-02664-s001.zip › Supplementary Table S3.pdf]

Table S3. Individual environments and combined overall BLUPs values for nine traits recorded in eight durum wheat tested across 15 environments.

Environment	Genotype	BLUP_TW	BLUP_GY	BLUP_HI	BLUP_TKW	BLUP_KA	BLUP_KW	BLUP_KL	BLUP_KC	BLUP_KLKW
IR-JU22	**Boabdil**	2022.027	747.528	37.231	51.118	19.936	3.496	7.801	NA	2.235
	**Bolenga**	2062.195	767.301	37.124	49.883	19.138	3.558	7.302	NA	2.070
	**Norsi**	2022.027	596.571	29.916	64.877	24.357	3.527	9.298	NA	2.631
	**NUR-072**	2148.555	800.002	36.452	62.255	23.127	3.435	9.231	NA	2.697
	**NUR-106**	2117.426	741.444	34.502	60.837	24.523	3.435	9.896	NA	2.895
	**NUR-238**	1996.922	586.684	29.904	49.239	19.238	3.404	7.768	NA	2.301
	**UC1113**	1984.370	733.839	37.592	44.606	18.207	3.282	7.635	NA	2.367
	**Um Qais**	2089.810	730.797	34.709	47.908	17.509	3.496	6.803	NA	2.004
**LSD(0.05)**		**145.595**	**73.388**	**3.384**	**1.499**	**0.453**	**0.078**	**0.158**		**0.098**

IR-KD20	**Boabdil**	1856.294	632.752	34.170	40.457	17.452	3.149	7.470	1.527	2.387
	**Bolenga**	1579.674	589.266	37.818	34.859	15.221	2.978	6.841	1.494	2.314
	**Norsi**	1500.113	434.209	30.537	53.696	22.023	3.205	9.164	1.719	2.881
	**NUR-072**	1982.387	707.687	35.248	57.826	22.809	3.264	9.260	1.699	2.855
	**NUR-106**	1610.606	638.309	39.878	58.904	24.283	3.408	9.665	1.729	2.848
	**NUR-238**	1809.137	722.203	39.146	56.493	21.322	3.546	8.041	1.471	2.278
	**UC1113**	1830.403	733.527	39.154	48.685	19.752	3.379	7.921	1.524	2.364
	**Um Qais**	1639.605	554.678	34.642	44.710	17.141	3.389	6.791	1.375	2.012
**LSD(0.05)**		**296.341**	**113.150**	**5.542**	**2.735**	**0.887**	**0.066**	**0.253**	**0.034**	**0.071**

IR-KD21	**Boabdil**	985.566	436.163	44.888	50.010	19.774	3.334	8.006	1.570	2.440
	**Bolenga**	1438.743	786.008	53.323	39.029	16.307	3.117	7.158	1.541	2.350
	**Norsi**	1023.903	303.042	30.621	54.023	22.943	3.334	9.245	1.716	2.769
	**NUR-072**	1311.309	560.241	42.691	61.198	23.241	3.427	9.082	1.629	2.650
	**NUR-106**	1199.021	609.799	50.300	59.712	25.552	3.520	9.799	1.774	2.889
	**NUR-238**	1056.205	424.942	40.633	54.939	21.425	3.489	8.266	1.541	2.380
	**UC1113**	1145.776	521.714	45.789	47.677	19.708	3.427	7.810	1.512	2.291
	**Um Qais**	1075.610	544.385	50.515	35.761	15.218	3.117	6.701	1.483	2.231
**LSD(0.05)**		**167.824**	**94.209**	**7.464**	**3.018**	**1.045**	**0.130**	**0.479**	**0.119**	**0.246**

Environment	Genotype	BLUP_TW	BLUP_GY	BLUP_HI	BLUP_TKW	BLUP_KA	BLUP_KW	BLUP_KL	BLUP_KC	BLUP_KLKW
**RF-JU20**	**Boabdil**	1299.949	395.821	26.900	37.615	16.898	3.043	7.477	1.571	2.479
	**Bolenga**	1517.917	570.691	31.314	28.341	14.043	2.827	6.675	1.529	2.395
	**Norsi**	2142.535	431.963	17.037	54.318	22.471	3.298	9.141	1.695	2.789
	**NUR-072**	1322.421	420.315	26.470	42.685	18.791	2.991	8.472	1.730	2.847
	**NUR-106**	1437.611	482.492	28.009	47.835	21.795	3.144	9.450	1.818	3.027
	**NUR-238**	1122.739	281.143	20.926	34.438	15.254	2.948	6.958	1.523	2.385
	**UC1113**	1291.595	395.156	25.378	29.282	15.111	2.857	7.198	1.610	2.551
	**Um Qais**	1678.530	578.039	28.693	34.522	14.765	3.030	6.592	1.497	2.251
**LSD(0.05)**		**116.114**	**49.799**	**2.690**	**1.234**	**0.426**	**0.066**	**0.144**	**0.059**	**0.124**
**RF-JU21**	**Boabdil**	908.374	320.349	35.990	31.829	15.842	2.863	7.503	1.670	2.702
	**Bolenga**	986.600	421.168	41.871	34.597	16.490	2.917	7.600	1.670	2.676
	**Norsi**	915.264	251.394	27.946	45.060	19.537	2.999	8.635	1.710	2.864
	**NUR-072**	947.848	289.609	30.679	34.240	16.976	2.808	8.117	1.730	2.891
	**NUR-106**	912.456	327.685	36.495	36.318	17.916	2.917	8.182	1.710	2.810
	**NUR-238**	985.443	367.158	36.753	30.557	14.902	2.754	7.244	1.650	2.676
	**UC1113**	875.549	246.996	29.277	27.710	14.513	2.808	6.953	1.630	2.541
	**Um Qais**	1035.184	463.678	43.525	26.699	13.022	2.699	6.565	1.630	2.541
**LSD(0.05)**		**100.858**	**45.999**	**4.711**	**2.411**	**1.051**	**0.140**	**0.365**	**0.094**	**0.199**
**RF-MR20**	**Boabdil**	873.779	256.191	29.156	34.193	15.746	2.996	7.131	1.561	2.421
	**Bolenga**	815.494	265.746	33.040	30.984	15.156	2.907	7.003	1.567	2.453
	**Norsi**	774.777	166.492	22.286	47.340	20.927	3.142	8.839	1.716	2.780
	**NUR-072**	939.904	312.402	32.380	36.729	17.998	2.861	8.695	1.862	3.050
	**NUR-106**	937.757	246.452	25.763	36.263	19.194	2.961	8.982	1.880	3.032
	**NUR-238**	813.676	191.570	23.924	29.677	15.606	2.820	7.574	1.707	2.726
	**UC1113**	845.129	250.432	29.726	28.040	14.984	2.836	7.274	1.659	2.613
	**Um Qais**	885.650	270.923	30.208	26.717	13.133	2.842	6.292	1.495	2.275
**LSD(0.05)**		**130.645**	**59.351**	**5.064**	**3.498**	**1.175**	**0.160**	**0.646**	**0.144**	**0.301**

Environment	Genotype	BLUP_TW	BLUP_GY	BLUP_HI	BLUP_TKW	BLUP_KA	BLUP_KW	BLUP_KL	BLUP_KC	BLUP_KLKW

**RF-MR21**	**Boabdil**	690.403	186.984	26.966	31.959	16.169	2.900	7.435	1.602	2.572
	**Bolenga**	777.279	222.393	28.485	26.441	13.526	2.755	6.646	1.569	2.479
	**Norsi**	784.385	187.264	24.010	43.935	19.769	3.045	8.685	1.698	2.852
	**NUR-072**	804.792	209.404	26.232	37.690	18.051	2.929	8.258	1.762	2.821
	**NUR-106**	796.642	195.567	24.584	36.696	20.099	3.016	9.014	1.795	3.007
	**NUR-238**	780.962	227.062	28.943	32.252	15.772	2.929	7.238	1.602	2.541
	**UC1113**	708.463	190.856	27.099	26.209	14.022	2.813	6.777	1.602	2.479
	**Um Qais**	772.324	201.011	26.046	27.141	13.559	2.813	6.580	1.537	2.417
**LSD(0.05)**		**92.433**	**33.809**	**3.964**	**2.922**	**0.780**	**0.120**	**0.333**	**0.055**	**0.177**

**RF-MR22**	**Boabdil**	908.706	227.216	24.397	33.879	16.017	2.906	7.470	NA	2.634
	**Bolenga**	880.870	226.888	25.566	31.723	14.503	2.906	6.743	NA	2.404
	**Norsi**	900.753	237.707	25.914	50.769	21.218	3.220	8.824	NA	2.765
	**NUR-072**	882.858	255.414	28.838	34.264	16.873	2.748	8.361	NA	3.093
	**NUR-106**	864.963	270.825	31.844	40.811	19.770	2.937	9.121	NA	3.125
	**NUR-238**	918.648	351.621	37.966	41.822	17.828	3.126	7.635	NA	2.470
	**UC1113**	821.220	225.250	28.450	37.335	17.136	3.094	7.569	NA	2.503
	**Um Qais**	918.648	262.629	27.928	37.766	15.754	3.063	6.677	NA	2.273
**LSD(0.05)**		**81.826**	**45.853**	**3.379**	**2.052**	**0.747**	**0.112**	**0.264**		**0.125**

**RF-MS20**	**Boabdil**	953.047	336.011	34.944	36.504	16.575	3.099	7.337	1.568	2.403
	**Bolenga**	946.096	366.671	38.349	32.713	15.156	3.005	6.844	1.505	2.338
	**Norsi**	945.464	183.624	19.813	52.090	21.162	3.255	8.652	1.631	2.630
	**NUR-072**	965.211	348.647	35.785	38.612	17.829	2.943	8.258	1.725	2.824
	**NUR-106**	1070.340	362.887	32.867	45.781	21.657	3.255	9.080	1.757	2.856
	**NUR-238**	785.045	226.441	30.528	39.031	16.740	3.099	7.272	1.505	2.338
	**UC1113**	785.045	266.395	35.184	31.014	15.651	2.943	7.206	1.600	2.468
	**Um Qais**	874.852	307.953	35.486	31.990	14.463	3.036	6.384	1.442	2.144
**LSD(0.05)**		**151.203**	**41.880**	**4.616**	**2.251**	**0.820**	**0.099**	**0.334**	**0.082**	**0.130**

**Environment**	**Genotype**	**BLUP_TW**	**BLUP_GY**	**BLUP_HI**	**BLUP_TKW**	**BLUP_KA**	**BLUP_KW**	**BLUP_KL**	**BLUP_KC**	**BLUP_KLKW**
**RF-MS21**	**Boabdil**	647.390	187.254	28.921	34.059	16.008	3.001	7.201	NA	2.434

	**Bolenga**	835.853	263.685	31.159	30.364	13.774	2.890	6.274	NA	2.203
	**Norsi**	528.772	65.155	12.301	36.422	18.736	3.028	8.327	NA	2.796
	**NUR-072**	672.619	216.405	32.040	42.273	18.440	3.084	7.962	NA	2.598
	**NUR-106**	555.804	144.729	26.007	42.433	19.525	3.028	8.591	NA	2.829
	**NUR-238**	585.222	180.059	30.873	37.473	16.501	3.028	7.168	NA	2.368
	**UC1113**	620.786	159.129	25.592	32.053	15.746	3.001	7.002	NA	2.368
	**Um Qais**	696.287	236.147	34.017	31.849	14.037	2.973	6.274	NA	2.137
**LSD(0.05)**		**87.986**	**42.504**	**5.090**	**2.949**	**0.779**	**0.077**	**0.222**		**0.084**

**RF-NS22**	**Boabdil**	1972.276	502.118	25.370	45.888	19.330	3.267	7.935	1.557	2.447
	**Bolenga**	1438.425	394.501	27.447	47.664	19.839	3.340	7.968	1.534	2.401
	**Norsi**	1398.881	238.037	17.219	51.831	22.938	3.328	9.359	1.726	2.839
	**NUR-072**	1998.132	500.591	25.041	48.778	21.119	3.142	9.114	1.769	2.929
	**NUR-106**	2156.310	504.865	23.503	54.993	24.761	3.340	9.984	1.792	3.005
	**NUR-238**	2084.826	586.837	27.933	46.951	19.323	3.300	7.868	1.527	2.391
	**UC1113**	1201.158	320.467	26.449	37.006	17.212	3.124	7.486	1.560	2.427
	**Um Qais**	1491.658	428.084	28.724	34.231	15.182	3.039	6.736	1.488	2.251
**LSD(0.05)**		**349.167**	**104.424**	**3.912**	**4.457**	**1.133**	**0.111**	**0.202**	**0.033**	**0.053**

**RF-RB20**	**Boabdil**	1024.728	467.852	44.598	34.435	16.560	2.965	7.488	1.597	2.491
	**Bolenga**	896.907	387.002	43.454	35.700	17.220	2.977	7.706	1.615	2.712
	**Norsi**	757.196	293.174	41.511	38.144	18.228	3.103	7.981	1.623	2.408
	**NUR-072**	871.951	261.495	30.160	37.258	18.041	2.919	8.467	1.806	2.995
	**NUR-106**	971.221	371.365	37.776	38.830	19.752	3.070	8.907	1.817	2.986
	**NUR-238**	899.879	338.422	37.751	35.254	16.743	2.984	7.540	1.594	2.539
	**UC1113**	950.412	413.621	43.180	34.746	16.692	2.972	7.528	1.603	2.542
	**Um Qais**	992.030	429.591	42.531	33.907	15.824	2.956	7.127	1.535	2.467
**LSD(0.05)**		**155.192**	**71.094**	**4.672**	**5.598**	**2.702**	**0.122**	**1.020**	**0.116**	**0.242**

**Environment**	**Genotype**	**BLUP_TW**	**BLUP_GY**	**BLUP_HI**	**BLUP_TKW**	**BLUP_KA**	**BLUP_KW**	**BLUP_KL**	**BLUP_KC**	**BLUP_KLKW**
**RF-RB21**	**Boabdil**	327.841	40.883	17.160	19.647	12.829	2.601	6.784	1.701	2.674
	**Bolenga**	346.842	104.686	18.140	18.570	11.263	2.415	6.281	1.642	2.644

	**Norsi**	321.051	40.251	9.001	21.165	14.171	2.570	7.666	1.820	2.972
	**NUR-072**	304.492	35.296	18.761	22.996	14.363	2.632	7.603	1.820	2.942
	**NUR-106**	307.410	52.287	12.325	24.689	15.962	2.694	8.169	1.880	3.061
	**NUR-238**	309.495	49.354	14.592	21.712	13.212	2.663	6.784	1.612	2.585
	**UC1113**	281.202	15.891	19.074	18.429	12.254	2.570	6.564	1.642	2.555
	**Um Qais**	310.567	49.632	29.054	27.518	13.980	2.787	6.816	1.582	2.466
**LSD(0.05)**		**47.795**	**16.109**	**3.148**	**2.475**	**0.905**	**0.091**	**0.478**	**0.118**	**0.233**

**RF-SK20**	**Boabdil**	893.714	207.313	11.929	36.913	17.446	3.090	7.535	1.573	2.452
	**Bolenga**	913.855	201.980	28.473	31.901	15.325	2.966	7.028	1.551	2.428
	**Norsi**	729.490	117.522	12.146	39.359	18.839	3.073	8.161	1.641	2.623
	**NUR-072**	872.106	246.878	11.744	38.211	18.332	3.055	8.102	1.641	2.648
	**NUR-106**	864.259	157.973	17.324	37.682	18.934	3.073	8.191	1.641	2.672
	**NUR-238**	750.077	194.422	16.027	33.086	16.465	3.020	7.326	1.573	2.452
	**UC1113**	674.057	213.095	5.869	30.368	15.578	3.002	7.147	1.573	2.452
	**Um Qais**	1083.689	231.761	16.221	35.757	15.515	3.055	6.909	1.506	2.306
**LSD(0.05)**		**69.399**	**33.426**	**3.336**	**3.862**	**1.069**	**0.120**	**0.554**	**0.104**	**0.236**

**RF-SK22**	**Boabdil**	944.303	155.561	23.425	30.601	15.765	2.888	7.267	1.568	2.503
	**Bolenga**	957.656	175.478	22.214	23.706	12.254	2.697	6.137	1.504	2.374
	**Norsi**	964.778	92.139	16.073	37.075	18.713	2.942	8.530	1.760	2.923
	**NUR-072**	969.229	189.630	28.224	33.977	16.825	2.806	7.965	1.696	2.858
	**NUR-106**	941.633	112.584	18.469	37.363	18.613	2.915	8.463	1.760	2.890
	**NUR-238**	963.888	145.602	25.886	30.271	14.771	2.861	6.901	1.536	2.438
	**UC1113**	944.838	173.905	31.333	27.526	14.407	2.833	6.735	1.504	2.374
	**Um Qais**	955.342	274.545	21.609	23.706	12.353	2.724	6.303	1.504	2.374
**LSD(0.05)**		**61.437**	**40.975**	**4.560**	**2.775**	**0.604**	**0.125**	**0.155**	**0.070**	**0.138**

**Environment**	**Genotype**	**BLUP_TW**	**BLUP_GY**	**BLUP_HI**	**BLUP_TKW**	**BLUP_KA**	**BLUP_KW**	**BLUP_KL**	**BLUP_KC**	**BLUP_KLKW**
**Overall(combined)**	**Boabdil**	1082.122	339.834	29.736	36.557	16.799	3.043	7.445	1.586	2.479
	**Bolenga**	1084.138	377.857	33.153	33.171	15.281	2.953	6.935	1.555	2.410

	**Norsi**	1062.932	249.996	22.452	45.989	20.424	3.138	8.715	1.711	2.777
	**NUR-072**	1105.204	355.802	29.371	41.960	18.874	3.005	8.486	1.749	2.862
	**NUR-106**	1093.983	346.670	29.292	44.029	20.893	3.113	9.070	1.791	2.947
	**NUR-238**	1071.479	327.448	29.527	38.147	16.986	3.060	7.429	1.566	2.453
	**UC1113**	1043.635	325.616	30.044	33.348	16.042	2.996	7.240	1.580	2.451
	**Um Qais**	1087.934	366.971	32.117	33.325	14.720	3.000	6.609	1.495	2.260
**LSD(0.05)**		**72.886**	**47.343**	**3.007**	**2.943**	**0.824**	**0.072**	**0.211**	**0.036**	**0.074**
