# Supplementary material for: Performance and Stability Analysis of Selected Durum Wheat Genotypes Differing in Their Kernel Characteristics
Source: Plants (Basel). 2023 Jul 16;12(14):2664. doi: 10.3390/plants12142664 (PMC10384256; doi:10.3390/plants12142664)
Supplement: Supplementary file 1 [file plants-12-02664-s001.zip › Supplementary Table S4.pdf]

Table S4. AMMI analysis of variance for GY, TKW and kernel-related traits in eight durum wheat genotypes tested across 15 different environments.

Variable GY

Source	Df	Sum Sq	Mean Sq	F value	Pr(>F)	Proportion (%)	Accumulated
**ENV**	14	11355146	811082	241.54	0	73.88	NA
**REP(ENV)**	30	100740	3358	2.3	0.000349	0.66	NA
**GEN**	7	679448	97064	66.38	8.5E-50	4.42	NA
**GEN:ENV**	98	1463940	14938	10.22	2.22E-44	9.52	NA
**IPC1**	20	602624	30131	20.61	0	41.2	41.2
**IPC2**	18	357469	19859	13.58	0	24.4	65.6
**IPC3**	16	199571	12473	8.53	0	13.6	79.2
**IPC4**	14	113939	8138	5.57	0	7.8	87
**IPC5**	12	98025	8169	5.59	0	6.7	93.7
**IPC6**	10	73196	7320	5.01	0	5	98.7
**IPC7**	8	19116	2390	1.63	0.118	1.3	100
**Residuals**	210	307085	1462	NA	NA	NA	NA
**Total**	457	15370298	33633	NA	NA	NA	NA

Variable TKW

Source	Df	Sum Sq	Mean Sq	F value	Pr(>F)	Proportion (%)	Accumulated
**ENV**	14	23857.7	1704.12	490.428	0	54.37	NA
**REP(ENV)**	30	104.2	3.47	0.877	0.654	0.24	NA
**GEN**	7	8986.3	1283.76 3	24.164	7.68E-109	20.48	NA
**GEN:ENV**	98	5050.9	51.54	13.014	6.6E-53	11.51	NA
**IPC1**	20	1783.7	89.19	22.52	0	35.3	35.5
**IPC2**	18	1477.3	82.07	20.72	0	29.2	64.6
**IPC3**	16	909	56.81	14.35	0	18	82.6
**IPC4**	14	478	34.14	8.62	0	9.5	92
**IPC5**	12	203	16.91	4.27	0	4	96
**IPC6**	10	145.3	14.53	3.67	0.0002	2.9	98.9
**IPC7**	8	54.7	6.83	1.73	0.093	1.1	100
**Residuals**	210	831.6	3.96	NA	NA	NA	NA
**Total**	457	43881.7	96.02	NA	NA	NA	NA

Variable KA

Source	Df	Sum Sq	Mean Sq	F value	Pr(>F)	Proportion (%)	Accumulated

**ENV**	14	1371.25	97.946	232.423	0	34.29	NA
**REP(ENV)**	30	12.64	0.421	0.877	0.654	0.32	NA
**GEN**	7	1741.52	248.788	517.858	1.06E-128	43.55	NA
**GEN:ENV**	98	386.43	3.943	8.208	3.77E-37	9.66	NA
**IPC1**	20	136.82	6.841	14.24	0	35.4	35.4
**IPC2**	18	120.6	6.7	13.95	0	31.2	66.6
**IPC3**	16	57.31	3.582	7.46	0	14.8	81.4
**IPC4**	14	31.38	2.241	4.67	0	8.1	89.6
**IPC5**	12	22.66	1.888	3.93	0	5.9	95.4
**IPC6**	10	12.95	1.295	2.7	0.0039	3.4	98.8
**IPC7**	8	4.72	0.59	1.23	0.283	1.2	100
**Residuals**	210	100.89	0.48	NA	NA	NA	NA
**Total**	457	3999.16	8.751	NA	NA	NA	NA

Variable KW

**Source**	**Df**	**Sum Sq**	**Mean Sq**	**F value**	**Pr(>F)**	**Proportion (%)**	**Accumulated**
**ENV**	14	15.9839	1.1417	193.05	0	62.15	NA

**REP(ENV)**	30	0.1774	0.00591	1.2	0.228	0.69	NA
**GEN**	7	1.6932	0.24188	49.12	6.55E-41	6.58	NA
**GEN:ENV**	98	3.4156	0.03485	7.08	1.51E-32	13.28	NA
**IPC1**	20	1.5496	0.07748	15.73	0	45.4	45.4
**IPC2**	18	0.6806	0.03781	7.68	0	19.9	65.3
**IPC3**	16	0.5276	0.03298	6.7	0	15.4	80.7
**IPC4**	14	0.3817	0.02726	5.54	0	11.2	91.9
**IPC5**	12	0.1782	0.01485	3.01	0.0006	5.2	97.1
**IPC6**	10	0.0512	0.00512	1.04	0.411	1.5	98.6
**IPC7**	8	0.0468	0.00585	1.19	0.306	1.4	100
**Residuals**	210	1.0341	0.00492	NA	NA	NA	NA
**Total**	457	25.7199	0.05628	NA	NA	NA	NA

Variable KL

**Source**	**Df**	**Sum Sq**	**Mean Sq**	**F value**	**Pr(>F)**	**Proportion (%)**	**Accumulated**
**ENV**	14	45.03	3.2164	45.985	2.22E-16	12.19	NA
**REP(ENV)**	30	2.098	0.0699	0.975	0.509	0.57	NA

**GEN**	7	257.173	36.7389	512.115	3.21E-128	69.63	NA
**GEN:ENV**	98	24.991	0.255	3.555	7.65E-15	6.77	NA
**IPC1**	20	11.083	0.5541	7.72	0	44.3	44.3
**IPC2**	18	5.574	0.3096	4.32	0	22.3	66.6
**IPC3**	16	3.83	0.2394	3.34	0	15.3	82
**IPC4**	14	2.682	0.1916	2.67	0.0013	10.7	92.7
**IPC5**	12	0.886	0.0738	1.03	0.422	3.5	96.3
**IPC6**	10	0.604	0.0604	0.84	0.591	2.4	98.7
**IPC7**	8	0.333	0.0416	0.58	0.794	1.3	100
**Residuals**	210	15.065	0.0717	NA	NA	NA	NA
**Total**	457	369.349	0.8082	NA	NA	NA	NA

Variable KC

**Source**	**Df**	**Sum Sq**	**Mean Sq**	**F value**	**Pr(>F)**	**Proportion (%)**	**Accumulated**
**ENV**	14	0.7736	0.05526	20.773	1.14E-11	11.53	NA
**REP(ENV)**	30	0.0798	0.00266	0.961	0.529	1.19	NA
**GEN**	7	4.0406	0.57722	208.587	7.08E-91	60.22	NA

**GEN:ENV**	98	0.6173	0.0063	2.276	0.000000375	9.20	NA
**IPC1**	20	0.283	0.01415	5.11	0	45.9	45.9
**IPC2**	18	0.1583	0.00879	3.18	0	25.6	71.5
**IPC3**	16	0.0712	0.00445	1.61	0.0682	11.5	83
**IPC4**	14	0.0435	0.0031	1.12	0.342	7	90.1
**IPC5**	12	0.0344	0.00287	1.04	0.413	5.6	95.6
**IPC6**	10	0.0153	0.00153	0.55	0.853	2.5	98.1
**IPC7**	8	0.0116	0.00145	0.52	0.841	1.9	100
**Residuals**	210	0.5811	0.00277	NA	NA	NA	NA
**Total**	457	6.7096	0.01468	NA	NA	NA	NA

Variable KLKW

**Source**	**Df**	**Sum Sq**	**Mean Sq**	**F value**	**Pr(>F)**	**Proportion (%)**	**Accumulated**
**ENV**	14	3.2724	0.23374	19.58	2.47E-11	10.22	NA
**REP(ENV)**	30	0.3582	0.01194	1	0.466	1.12	NA
**GEN**	7	19.6568	2.80812	236.23	7.35E-96	61.40	NA
**GEN:ENV**	98	3.1146	0.03178	2.67	1.46E-09	9.73	NA

**IPC1**	20	1.3337	0.06668	5.61	0	42.8	42.8
**IPC2**	18	0.9338	0.05188	4.36	0	30	72.8
**IPC3**	16	0.3162	0.01977	1.66	0.0565	10.2	83
**IPC4**	14	0.2314	0.01653	1.39	0.16	7.4	90.4
**IPC5**	12	0.1507	0.01256	1.06	0.396	4.8	95.2
**IPC6**	10	0.0955	0.00955	0.8	0.629	3.1	98.3
**IPC7**	8	0.0532	0.00665	0.56	0.81	1.7	100
**Residuals**	210	2.4963	0.01189	NA	NA	NA	NA
**Total**	457	32.0129	0.07005	NA	NA	NA	NA
